# Microbial Sterolomics as a Chemical Biology Tool

**DOI:** 10.3390/molecules23112768

**Published:** 2018-10-25

**Authors:** Brad A. Haubrich

**Affiliations:** Department of Chemistry, University of Nevada, Reno, Reno, NV 89557, USA; bhaubrich@unr.edu; Tel.: +1-775-784-4857

**Keywords:** algal sterols, ergosterol biosynthesis, infectious disease, lipidomics, oxyphytosterol, pharmacognosy, phytosterol, sterolomics

## Abstract

Metabolomics has become a powerful tool in chemical biology. Profiling the human sterolome has resulted in the discovery of noncanonical sterols, including oxysterols and meiosis-activating sterols. They are important to immune responses and development, and have been reviewed extensively. The triterpenoid metabolite fusidic acid has developed clinical relevance, and many steroidal metabolites from microbial sources possess varying bioactivities. Beyond the prospect of pharmacognostical agents, the profiling of minor metabolites can provide insight into an organism’s biosynthesis and phylogeny, as well as inform drug discovery about infectious diseases. This review aims to highlight recent discoveries from detailed sterolomic profiling in microorganisms and their phylogenic and pharmacological implications.

## 1. Introduction

**Figure 1:**
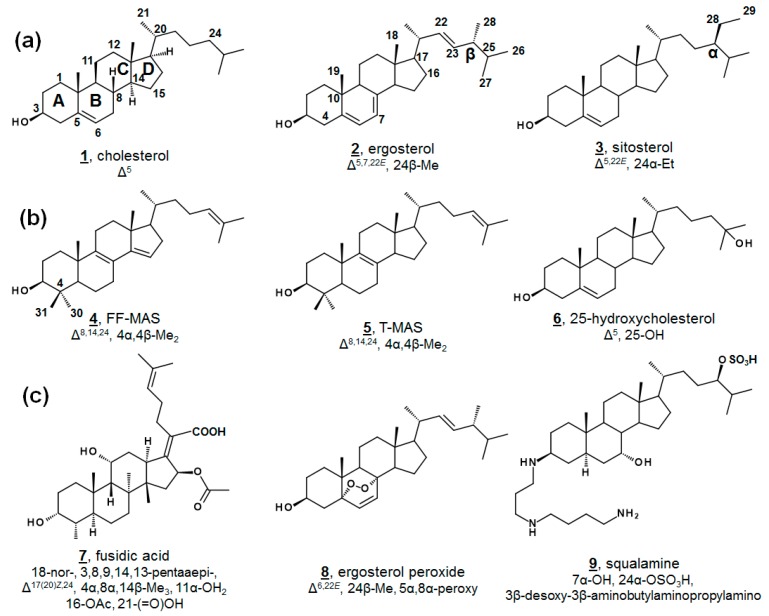
Structure and numbering systems of sterols and steroids. (**a**) Δ^5^ end product inserts from mammals, fungi, and vascular plants, respectively, cholesterol **1**, ergosterol **2**, and sitosterol **3**. (**b**) Examples of steroidal metabolites important in human biology for F-MAS **4**, TT-MAS **5**, 25-hydroxycholesterol **6**. (**c**) Examples of steroidal metabolites from nonhuman sources with bioactivity, fusidic acid **7**, ergosterol peroxide **8**, and squalamine **9**. The numbering system shown here, and used in this manuscript, is the conventional system [1]. Designations of α and β within the sterol nucleus signify below and above the plane. Unrelated to nucleus α and β, substituents on C24 are also designated α and β to reflect the C24 stereochemistries of sitosterol and ergosterol, respectively, as drawn above. Carbon numbering is provided on 1–4, and stereochemistries at C8, C9, C14, and C16 on structure **1** are hereafter implied on structures, unless otherwise annotated as in fusidic acid. Molecular features for each structure are provided relative to 5α-cholestanol for clarity. For a complete list of systematic names of compounds, see Table A1.

Sterols, like cholesterol **1**, ergosterol **2**, and sitosterol **3**, as well as secondary metabolites, are amphipathic lipids that contain a 1,2-cyclopentanoperhydrophenanthrene ring nucleus (Figure 1). Sterols are ubiquitous molecules found in all eukaryotic life, serving a multitude of crucial biological functions [1]. Some prokaryotes synthesize sterols as well, and some prokaryotes contain enzymes with incomplete Δ^5^ sterol biosynthesis [1,2,3,4,5]. While sterol biosynthesis may predate eukaryotes [6], it is often hypothesized that aside from the protomitochondrial lineage, most bacteria have gained these genes via lateral gene transfer [3,4]. The end product of Δ^5^ sterols such as cholesterol **1** and ergosterol **2** (Figure 1a) contribute to cell membrane fluidity in their bulk insert role in mammals and fungi, respectively [1,7]. Steroidal secondary metabolites of the steroid hormone and bile acid classes serve well-known important roles in inflammation, sex characteristics, and lipid absorption [7].

Minor components within the human sterol metabolome, which serve unusual but essential functions, have also been identified. For instance, the meiosis-activating sterols (MASs) 4,4-dimethylcholesta-8(9),14(15),24-trienol (follicular fluid meiosis-activating sterol; FF-MAS) **4** and 4,4-dimethylcholesta-8(9),24-dienol (testicular meiosis-activating sterol; T-MAS) **5** are biosynthetic intermediates in the cholesterol pathway that signal meiosis in mammalian oocytes and spermatozoa [7]. Various minor metabolites occurring both upstream and downstream of cholesterol have been demonstrated as ligands for nuclear hormone receptors and play critical roles in development and immunology, including 25-hydroxycholesterol **6** (Figure 1b) [8,9,10,11]. Recent advances in methodologies in lipidomics have expedited discoveries with regard to these necessary minor human sterols and steroids, as well as provided new diagnostic screens for patients with dysregulated sterol biosynthesis, as in Niemann-Pick and Smith-Lemli-Opitz syndrome. Contemporary discoveries in human sterolomics [11,12,13,14], as well as plant sterolomics [15], have been reviewed extensively elsewhere.

Metabolites can also be used to classify organisms and explore evolutionary relationships. Sterol distribution has long been used for chemotaxonomic purposes in plants [16], fungi [17,18,19], and other microorganisms [20,21,22]. There is also potential for sterols to serve as biomarkers, and sterol composition can play a role in feedstocks.

**Figure 2:**
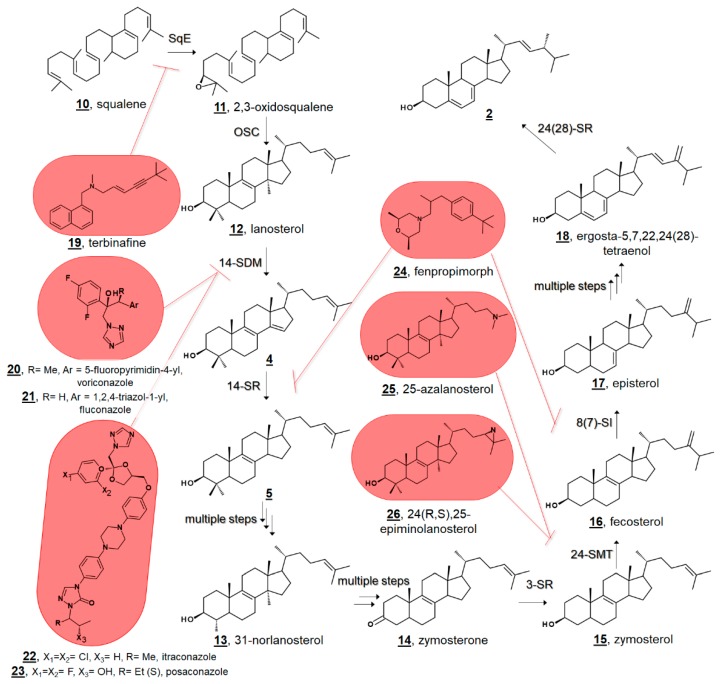
Truncated hypothetical pathway of fungal ergosterol **2** biosynthesis from squalene **10**. Inhibitor targets of squalene epoxidase (SqE) by allylamines, e.g., terbinafine **19**, sterol C14-demethylase (14-SDM = CYP51) by azoles, e.g., voriconazole **20**, fluconazole **21**, itraconazole **22**, and posaconazole **23**, sterol C14-reductase (14-SR) and sterol C8(7)-isomerase (8(7)-SI) by morpholines, e.g., fenpropimorph **24**, and sterol C24-methyltransferase (24-SMT) by 25-azalanosterol **25** or 24(R,S),25-epiminolanosterol **26** are highlighted at the biosynthetic steps they block. 3-SR; sterol C3 reductase, 24-SR, sterol C24 reductase.

Small molecule ligands for ergosterol biosynthetic enzymes in fungi have long been clinically and agriculturally relevant [23,24,25]. Marketed antimycotics include molecules in such classes as allylamines, which target squalene epoxidase (SqE); azoles, which target sterol C14-demethylase (14-SDM = CYP51, =Erg11p in fungi); and morpholines, which target both sterol C14-reductase (14-SR, =Erg3p in fungi) and sterol C8(7)-isomerase (8(7)-SI, =Erg2p in fungi) (Figure 2) [24]. There is further interest in the design and discovery of inhibitors of other sterol enzymes, particularly sterol C24-methyltransferase (24-SMT, =Erg6p in fungi), which is absent from humans’ cholesterol biosynthesis [1,25,26]. Understanding sterol biosynthesis in non-fungal microbes may provide new insights for treating infections by eukaryotic pathogens.

Novel metabolites isolated from microbial sources are conversely often found to exhibit biological activity. Famously, fusidic acid **7** (Figure 1c), originally isolated from fungal *Fusidium* spp., is a tetracyclic triterpene antibacterial and has been used in the clinic for decades [27,28,29]. Fusidic acid inhibits growth by restricting protein synthesis via elongation factor G in Gram-positive bacteria, including *Streptococcus* spp., *Clostridium* spp., and penicillin-resistant strains of *Staphylococcus* spp. [28,29]. Structural analogues of fusidic acid, have shown varying antimicrobial, as well as anticholesterolemic and antineoplastic, characteristics [29]. Isolated from a variety of fungi and sponges, as well as vascular plants, ergosterol peroxide **8** possesses broad bioactivity, including anti-tumor, immunomodulatory, inhibitory hemolytic, anti-inflammatory, antioxidant, and antimicrobial properties. Several other endoperoxides of other phytosterols and of cholestenols have been reported to have similar properties, as well [30,31,32,33,34]. Squalamine **9** is a non-microbially derived natural steroidal, which has demonstrated antimicrobial and antiangiogenic properties and has led to interest in synthetic analogues for structure-activity improvement [35].

This short review aims to highlight new findings in microbial sterolomics, with respect to phylogeny, ecology, biosynthesis for drug discovery, and discovery of bioactive metabolites.

## 2. Phylogenic and Ecological Insights

### 2.1. Algal Phytosterol Biosynthesis

**Figure 3:**
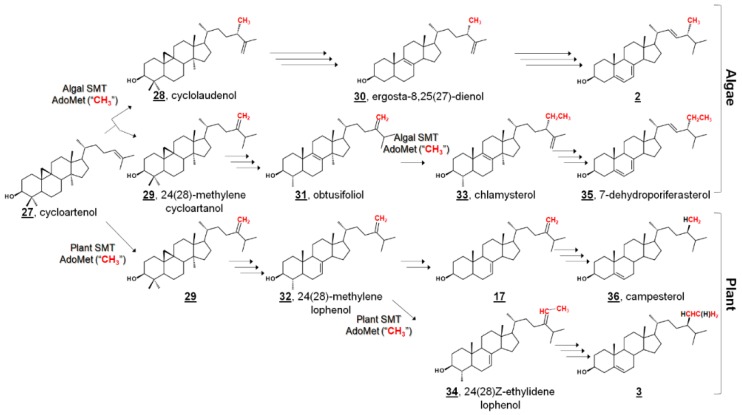
Comparative phytosterol biosynthesis in the photosynthetic lineage from the protosterol cycloartenol **27**. In algae, 24-methyl and 24-ethyl sterols arise from a bifurcation of products of biomethylation by sterol methyltransferase (SMT); In higher plants, they arise from alternate pathways from the intermediate 24(28)-methylene lophenol **30**, which can be methylated again or metabolized to campesterol **36**. Red methyl groups from SMT co-substrate S-adenosyl methionine (AdoMet) are annotated to show hypothetical labeling patterns of Δ^5^ sterols as discussed in [45,50]. An additional 15 algal sterols were reported in [45]. Truncated fungal phytosterol biosynthesis from protosterol lanosterol **12** is illustrated in Figure 2.

Ergosterol **2**, having long been considered the “fungal sterol”, is nevertheless present in every major eukaryotic kingdom [1]. Ergosterol is present in amoebae [21,22,36,37] and trypanosomatids [38,39,40,41,42,43,44], and ergosterol is a major sterol of many taxa within green algae [20,45,46,47,48]. The unicellular green alga model organism *Chlamydomonas reinhardtii* uses ergosterol and its 24-ethyl analogue, 7-dehydroporiferasterol **35**, as its main Δ^5^ sterols [45,46]. Vascular plants, on the other hand, chiefly use campesterol **36** and sitosterol **3** as Δ^5^ membrane inserts (Figure 3) [1,49]. Ergosterol and 7-dehydroporiferasterol differ from campesterol and sitosterol by units of unsaturation (double bonds) in the sterol nucleus and side chain, as well as stereochemistry at C24. While all four compounds possess 24*R* stereochemistry, 24-alkylation of ergosterol and 7-dehydroporiferasterol has β-stereochemistry (alkyl groups behind the plane, as drawn), while 24-alkylation of campesterol and sitosterol has α-stereochemistry (above the plane, as drawn) [1,45]. Conversely, the green alga synthesizes sterol from the photosynthetic protosterol. Fungi (nonphotosynthetic lineage) cyclize 2,3-oxidosqualene to lanosterol (Figure 2), while higher plants, and green algae, (photosynthetic lineage) cyclize 2,3-oxidosqualene to the plant protosterol cycloartenol [1,45].

In *C. reinhardtii*, the biochemical pathway from the “plant” protosterol cycloartenol to the “fungal” Δ^5^ end product was investigated by sterolomic experiments of *C. reinhardtii* cultures. Sterol profiling of wild-type, mutant, and inhibitor-treated cultures revealed an additional 21 sterols beyond cycloartenol, ergosterol, and 7-dehydroporiferasterol **33** [45] (Figure 3). *C. reinhardtii* cultures that were not treated with a 24-SMT inhibitor contained only cycloartenol and 24-alkylsterols, indicating that bioalkylation and introduction of C28 by algal 24-SMT occurs upon cycloartenol itself early in the pathway. Further, 24-methylated cycloartenols were 24β-methylcycloart-25(27)-enol (cyclolaudenol) **28** and 24(28)-methylenecycloartanol **29**, signifying a bifurcation of methylated products of algal 24-SMT [45]. The presence of C29 (i.e., a 24-ethyl group) on a 4α,14α-dimethyl sterol **33** led to the identification of obtusifoliol **31** as the substrate for the second biomethylation reaction of the algal sterol side chain, different from the substrate preference in higher plants (Figure 3). Furthermore, the alkylation product in plants has a 24-ethylene substituent, whereas the product in *C. reinhardtii* was found to bear a 24β-ethyl group with desaturation at C25 [45].

This pathway delineates algal biosynthesis of ergosterol disparate from the fungal pathway. In the former Δ^25(27)^-olefin pathway, *C. reinhardtii* alkylates sterols at C24 in a bifurcated manner to Δ^25(27)^-olefin and Δ^24(28)^-olefin products. Δ^24(28)^-Olefin products are further metabolized and later alkylated at C28 to only 24β-ethyl-Δ^25(27)^-olefin products. Conversely, fungal bioalkylation of C24 yields only Δ^24(28)^-olefin products, which are reduced to eventually yield ergosterol. That is, the stereochemistry of C24 in algal ergosterol arises from the methylation steps, whereas the stereochemistry of C24 in fungal ergosterol arises from a successive reduction step [45]. The Δ^25(27)^-olefin pathway was confirmed by sterol profiling of cultures incubated with isotopically labeled [methyl-^2^H_3_]methionine ([^2^H_3_]Met). These algal cultures incorporated three and five deuterium atoms into ergosterol and 7-dehydroporfierasterol, respectively [45].

The algal pathway was further corroborated by characterization of recombinant *C. reinhardtii* 24-SMT, found to catalyze the methylation of C24 by introduction of C28 and the methylation of C28 with C29. *C. reinhardtii* 24-SMT favored cycloartenol as a substrate, and a bifurcation of products to cyclolaudenol **28** and 24(28)-methylenecycloartanol **29** was found in ratios comparable to in vivo ratios of ergosterol and 7-dehydroporiferasterol [50]. A switch to Δ^25(27)^-olefin “algal” products of fungal or plant 24-SMT has been noted upon mutagenesis or incubation with electronically modified substrates [49,51]. In addition, obtusifoliol was found to be a substrate for the second methyltransfer of *C. reinhardtii* 24-SMT, 24β-methyl-Δ^25(27)^-sterols were not substrates, and incubation with [methyl-^2^H_3_]S-adenosyl methionine (^2^H_3_-AdoMet) produced labeled products with three and five deuterium atoms [50].

**Figure 4:**
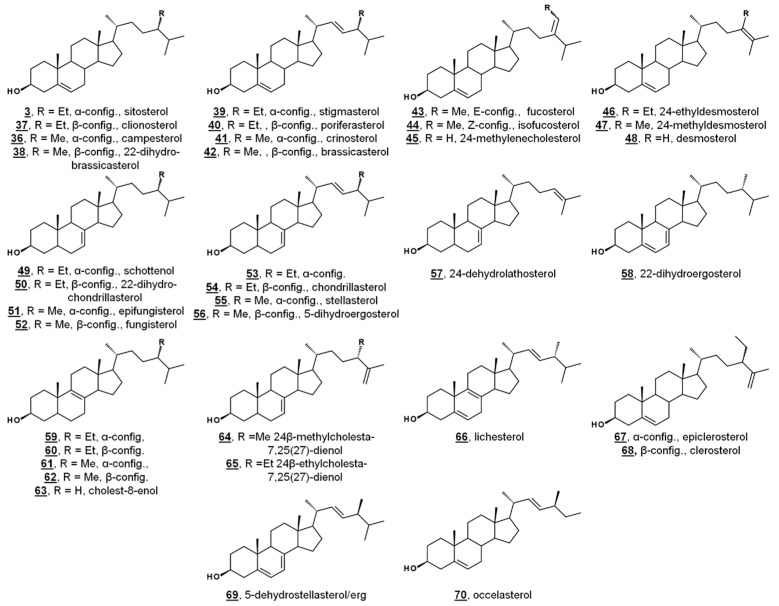
Molecular structures of algal sterols.

Green algae from the *Acicularia* spp. and *Acetabularia* spp. are macroscopic, yet unicellular. With a long and uninterrupted fossil record, they are often used to provide insight into the evolution of green algae and plants. Δ^5^-Bulk sterols of these genera lack Δ^7^ desaturations, in contrast to Chlamydomonas. Trimethylsilylated (TMS) sterols extracted from *Acicularia schenckii* and four species of Acetabularia revealed a principal Δ^5^ sterol (60–70%) of 24-ethylcholesterol (24α/24R = sitosterol **3**, 24β/24S = clionosterol **37**). Four other minor Δ^5^ sterols occurred in all five species: 24-methylcholesterol (24α/24R = campesterol **36**, 24β/24S = 22-dihydrobrassicasterol **38**), 24-ethylcholesta-5,22*E*-dienol (24α/24S = stigmasterol **39**, 24β/24R = poriferasterol **40**), 24-methylcholesta-5,22*E*-dienol (24α/24S = crinosterol **41**, 24β/24R = brassicasterol **42**), and 24-ethylidenecholesterol, which was tentatively assigned by the authors as the Δ^24(28)Z^ isomer = isofucosterol **44**. Among the TMS-derivatized sterols of *Acetabularia caliculus*, 24-ethylcholest-7-enol **46**/**47** was identified (Figure 4, Table 1) [52]. Prior studies had also identified cholesterol and 24-methylenecholesterol **45** in cultures of *Acetabularia mediterranea*, suggested by the authors to potentially be a result of differences in algal cultivation. *Acetabularia caliculus* also contained 24-ethylcholesterol in the sterol ester fraction, while the other Acetabularia species and *Acicularia schenckii* did not contain sterols in the ester fraction. These nearly identical sets of sterols from the five species, with a large separation in their geographical origin, illustrate a lack of divergence in sterol composition. It was thus hypothesized that these sterols represent an ancient biochemical trait within the photosynthetic lineage [52].

A study investigating the sterolome via free sterols and TMS derivatives from various classes of microalgae showed two species of the green algae Chlorella, *C. vulgaris* and *C. luteoviridis*, possessing different sterol profiles [53]. *C. vulgaris* contained chiefly ergosterol and fungisterol **52**. In the past, *C. vulgaris* has been reported to also contain 7-dehydroporiferasterol. The reported minor components included 5-dihydroergosterol **56**, 22-dihydroergosterol **58**, 24β-methylcholesta-7,25(27)-dienol **64**, 24β-methylcholest-8(9)-enol **62**, and lichesterol **66**. Conversely, the profile of *Chlorella luteoviridis* was dominated by poriferasterol **40** and 22-dihydrobrassicasterol **38**, with minor composition by clionasterol **35**, brassicasterol, and fungisterol [53]. The predominant sterol from *Nanochloropsis limnetica* was cholesterol, while its minor components were isofucosterol **44**, 24-ethylcholesterol (**3**/**37**), 24-methylenecholesterol **45**, and clerosterol **68** [53]. This report included the sterol profiling of several species of diatoms. The diatom *Stephanodiscus hantzschii*, whose sterols had not been studied prior to this report, had a composition of mostly 24-methylenecholesterol, with minor components of desmosterol **48**, 24-methylenelathosterol (Δ^7^, rather than Δ^5^, termed episterol above) **17**, and traces of two other sterols. Sterols from diatoms *Cyclotella meneghiniana* and *Gomphonema parvulum* were analyzed, with principal sterols of 24-methylenecholesterol and epibrassicasterol (called crinosterol, above; for list of trivial and systematic names, see Table A1) **41**, respectively. *C. meneghiniana* also contained desmosterol **48**, 24-methylenelathosterol, 24-dehydrolathosterol **57**, and 24-ethyldesmosterol, and *G. parvulum* contained 5-dehydrostellasterol/ergosterol **69**/**2**, 24α/β-ethylcholest-8(9)-enol **58**/**59**, and campesterol/22-dihydrobrassicasterol **36**/**38** (C24 alkyl group was presumably α-oriented) [53]. A brief list of recently reported algal sterols by taxonomic class is presented in Figure 4 and Table 1; for more comprehensive and historical lists, see Refs. [47,48].

### 2.2. Trophic and Limnological Sterols

**Figure 5:**
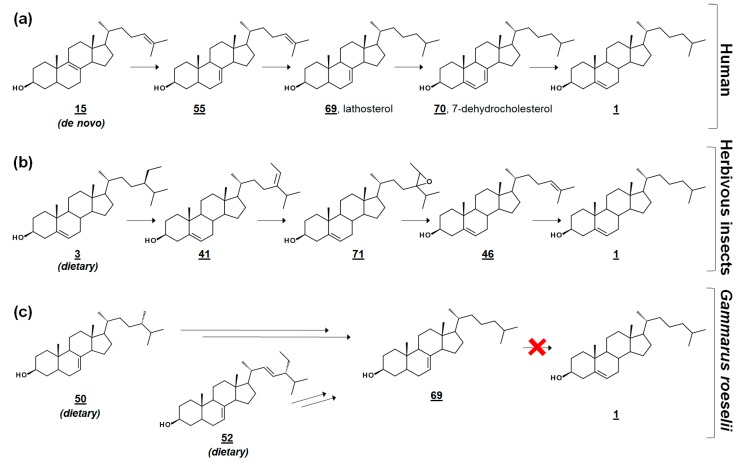
Comparative cholesterol biosynthesis between humans and arthropods. (**a**) Late-stage cholesterol biosynthesis in humans from *de novo* zymosterol **15**. (**b**) Proposed synthesis of cholesterol in herbivorous insects via dealkylation of dietary plant sterols (sitosterol) [58]. (**c**) Amphipod *Gammarus roeselii* can dealkylate the side chain of Δ^7^ algal sterols, such as fungisterol and chondrillasterol, but cannot produce cholesterol [56].

In the cross-class algal study [53], the researchers presented these profiles, along with their quantification, as references to the algal sterolome. As prey, Δ^7^ and Δ^7,22^ sterols are often nutritionally inadequate to invertebrate consumers [53,56,57]. Many invertebrates are auxotrophic for sterols and rely on diet to fulfill their sterol needs for cell membrane and hormonal requirements. Several of these specimens contain alternate enzymes, which dealkylate side chains of phytosterols, yet they lack the enzymes to desaturate C5–C6 or reduce C7–C8 (Figure 5) [57,58]. It has been proposed that these quantitated algal sterolome references can be used for studies involving the nutritional content of aquatic microorganisms for aquatic invertebrates [53]. Another study monitored the sterol profiles of an algal diet and the amphipod consumer *Gammarus roeselii*. Prey alga *N. limnetica*, rich in cholesterol, and alga *S. obliquus*, lacking cholesterol but rich in Δ^7^ sterols (See Table 1), were fed to *G. roeselii*. The sterol profile of *S. obliquus*-fed *G. roeselii* decreased in cholesterol, and increased in the Δ^7^ metabolite lathosterol **69**, detectable when the diet was 50% *S. obliquus* (Figure 5) [56].

Isotopically labeled sterolomic experiments have been used to explore trophic modifications by the Northern Bay scallop *Argopecten irradians irradians*. Dietary alga Rhodomonas was supplemented with sterols enriched with ^13^C at the C22 position. The ^13^C-label was noted on new sterol metabolites, including those newly desaturated with Δ^7^ and those bearing an introduced 4α-methyl group. The mollusk’s ability to synthesize cholesterol from food was noted to correlate to Δ^5^ double bonds in the dietary sterols. They were more likely to dealkylate side chains possessing 24-ethyl groups. The only 24-methyl sterols dealkylated by *A. irradians* contained a Δ^24(28)^ olefin (i.e., 24-methylene, rather than 24-methyl) [55].

A recent study investigated the lipid content of 37 strains within 10 classes of phytoplankton. Four classes, Cryptophyceae, Chlorophyceae, Treouciophyceae, and the diatoms are additionally represented in Table 1; this study additionally included dinoflagellates, euglenoids and the conjugatophyceae. Of the 37 strains, 29 sterols were detected, with notable variability of profile as a function of taxonomic class. The authors suggested Δ^5,22^ sterols as a potential biomarker for Chlorophyceae *Sphaerocystis* sp. and ergosterol as a potential biomarker for *Chlamydomonas* in habitats lacking other aquatic ergosterol-synthesizing microorganisms [59].

**Figure 6:**
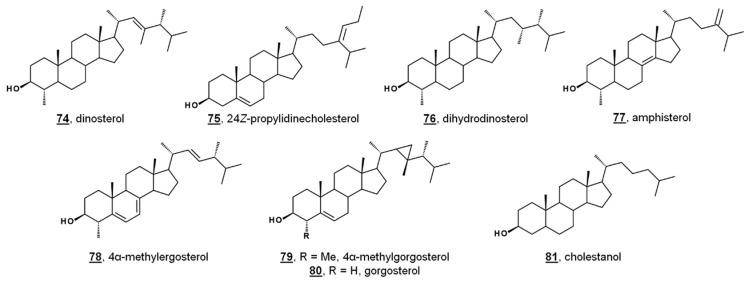
Sterol structures from various dinoflagellates.

While sterol metabolites of toxic blooms are likely non-toxic to fish populations, these metabolites may have a stronger influence on marine invertebrates. Toxic bloom-causing algae *Chloromorum toxicum*, *Chattonella marina*, *Heterosigma akashiwo*, and *Verrucophora farcimen* [54] have sterol profiles given in Table 1. *Verrucophora* sp. were found to produce the rare 27-nor sterol occelasterol **68** (Figure 4) [54]. It has been proposed that isofucosterol **44** is a potential biomarker for the green-tide forming multicellular alga *Ulva prolifera*, and that dinosterol **74** and 24*Z*-propylidienecholesterol **75** are potential biomarkers for bloom-forming dinoflagellates [60] (Figure 6). Toxic bloom-causing dinoflagellate *Cochlodinium polykrikoides* had a sterol profile including prevalent sterols of dinosterol **74** (40%), dihydrodinosterol **76** (32%), and the rare 4α-methyl sterol amphisterol **77** (23%). Small amounts of 4-methylergost-24(28)-enol **78** (5%) were detected [61]. Two isolates of the bioluminescent dinoflagellate *Pyrodinium bahamense* had a sterol profile of largely cholesterol (74–75%), but also components of dinosterol **74** (13–14%) and 4α-methylgorgosterol **79** (11–13%), analyzed as their TMS derivatives. 4α-Methylgorgosterol is uncommon in dinoflagellates and has potential as a biomarker (Figure 6) [62].

Lipidomic study of the coral *Dendrophyllia cornigera* revealed a geographical correlation to diet. *D. cornigera* analyzed from the Cantabrian Sea in the Northeast Atlantic reflected a productive environment, and the coral contained a high diversity of phytosterols. *D. cornigera* sampled from the Menorca Channel in the Mediterranean had a lower sterol content per dry weight and had less phytosterols. The Mediterranean coral had a higher relative abundance of occelasterol **70**, brassicasterol **42**, and cholestanol **81**, or cholesterol and ergosterol, depending on the sample. The difference in the geographic profiles was attributed to a diet high in phytoplankton and herbivorous grazers in the Cantabrian coral, and a diet primarily consisting of dinoflagellates in the Mediterranean coral [63]. Specimens of the coral *Agaricia* spp. taken from shallow waters and deep waters were found to have markedly different sterol profiles from one another. From shallow Caribbean waters, *Agaricia* contained mostly cholesterol and 24-methylenecholesterol, with lower abundances of other phytosterols. Samples from deep waters contained mostly cholesterol and 24-ethylcholesterol. No gorgosterol was detected in either set. The Caribbean coral *Montastraea cavernosa* contained mostly 24-methylcholesterol, followed by cholesterol and gorgosterol, and variation in subsurface depth did not cause a significant change in sterol content. It was concluded that *Agaricia* spp. relies primarily on heterotrophy, even at greater depths [64].

## 3. Sterolome-Informed Antimicrobial Targets

### 3.1. Trypanosoma Brucei

Trypanosomatids are flagellated protozoa, all of which are parasitic. Some examples from this clade are *Crithidia fasciculata*, solely parasitic to insect hosts, *Phytomonas serpens*, soley phytopathogenic, and a number of human pathogens, including *Trypanosoma cruzi*, *Leishmania* spp., and *Trypanosoma brucei*, which are the etiological agents of the following human diseases: leishmaniasis, Chagas’ disease, and human African trypanosomiasis (also known as African sleeping sickness), respectively. Most of the species, *C. fasciculata* [38], *P. serpens* [40], *T. cruzi* [38,44], and *Leishmania* spp. [39] synthesize ergosterol and other 24β-methyl/24(28)-methylene-sterols (ergostenols) *de novo* as their Δ^5^ end products. In light of this de novo biosynthesis, there has been interest in using ergosterol biosynthesis inhibitors (EBIs) to treat Chagas’ disease and leishmaniasis, and some molecules have even progressed to the clinic [25,44]. *Trypanosoma brucei*, conversely, synthesizes ergostenols during its life cycle in the insect vector (procyclic form (PCF)), but uses largely cholesterol from the host’s blood as its Δ^5^ bulk sterol in the human host (bloodstream form (BSF)) [41,42,43].

**Figure 7:**
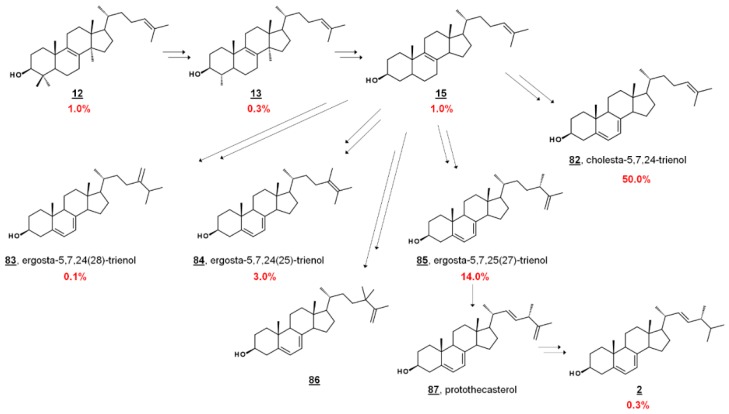
Abbreviated biosynthetic sterol pathway and composition in *T. brucei*. In *T. brucei*, C4 is demethylated before C14, contrary to mammalian and fungal pathways (cf. Figure 2). Values are percentage sterol composition reported by Zhou et al. [43]. Dietary cholesterol **1** accounted for 20.0 %, and other components were **16** (0.1%), **30** (1.0%), **48** (1.0%), **57** (8.0%), and others (0.2%). 24,24-Dimethylcholesta-5,7,25(27)-trienol and **86** and protothecasterol **87** were not detected in this composition, but have been reported in subsequent studies [42,66], respectively.

In the fly vector, cholesterol comprises a significant portion of the PCF sterol content. The profile contains sterols endogenous to PCF *T. brucei*, including prominent cholesta-5,7,24-trienol **82** and ergosta-5,7,25(27)-trienol **85**. PCF synthesizes trace ergosterol **2**; Ergosta-5,7,24(28)-trienol **85** and ergosta-5,7,24(25)-trienol **84** comprise some of the minor compounds present [41,42,43] (Figure 7). 24,24-Dimethylcholesta-5,7,25(27)-trienol **86** has also been detected in PCF profiles [42]. Culturing PCF in lipid-depleted media yields a higher composition of endogenous ergostenols and cholesta-5,7,24-trienol **82** relative to cholesterol [42,43]. Treatment of PCF cells with the 24-SMT inhibitor 25-azalanosterol **25** causes an increase in cholestenols in the profile [43].

Sterolomic analysis of PCF revealed a novel biosynthetic network. For instance, T. brucei demethylates protosterol lanosterol **12** at C4 initially (Figure 7), compared to mammalian and fungal pathways demethylating C14 first (cf. Figure 2) [42,43]. Moreover, the side chain methylation patterns of 24-SMT to yield Δ^24(28)^, Δ^25(27)^, and Δ^24(25)^ products, as well as the Δ^25(27)^ 24,24-dimethyl product **86**, are unique [42,43,65]. Isotopic experiments with ^13^C-labeled carbon sources leucine, acetate, and glucose were shown to produce variable labeling of Δ^5^ endproducts and biosynthetic intermediates. No labelling was noted on cholesterol. Isotopic incorporation was higher with acetate and glucose. The variability of labeling was potentially attributed to the equilibrium of acetyl-CoA pools in the mitochondria and cytosol [42]. Trypanosomal sterols protothecasterol **87** (ergosta-5,7,22*E*,25(27)-tetraenol), cholesta-5,7,24-trienol **82**, and ergosta-5,7,25(27)-trienol **83** have also been noted to incorporate isotope labeled from threonine [66].

In BSF *T. brucei*, however, the sterol content is overwhelmingly cholesterol, as well as dietary phytosterols, like sitosterol **3** and campesterol **36**, present in the hosts’ blood [41,42,43]. Single trace ^13^C-labeled sterol was found in BSF cultures fed [1-^13^C]glucose [42]. Upon removal of the main sterol component cholesterol, detailed targeted sterolomics of BSF *T. brucei* cells revealed minor components of the sterol profile. Due to the *S*-cis double bond configuration in the B ring of ergosterol and compounds **81**–**87**, UV absorbances of 282 nm can be monitored for the presence of endogenous Δ^5,7^ sterols, absent in serum. Endogenous cholesta-5,7,24-trienol and ergostenols were found at trace amounts, while they were undetectable when the presence of cholesterol was predominant. The ergosterol requirements for BSF was estimated to be 0.01 fg/cell, compared to the PCF requirement of 6 fg/cell [41]. Consequently, treatment with the EBIs itraconazole **22** and 25-azalanosterol **25** resulted in parasite death and an increased survival rate of infected mice. Correspondingly, the effects of EBIs on cultures were reversed upon supplementation of ergosterol [41].

**Figure 8:**
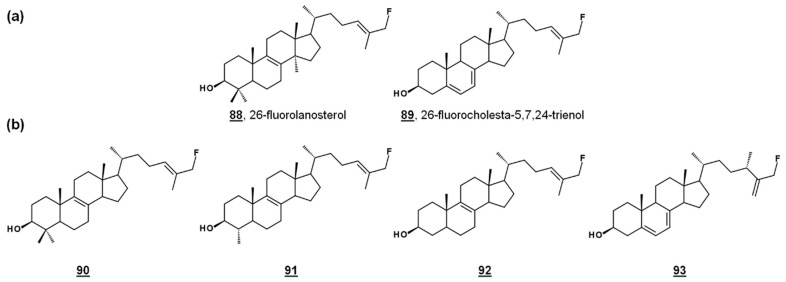
26-Fluorinated sterol analogues. (**a**) Fluorinated inhibitors of *T. brucei* 24-SMT and growth. (**b**) Metabolites of **88** identified from *T. brucei* and HEK cells [67].

24-SMT substrate analogues substituted with fluorine at C26, **88** and **89** (Figure 8a), inhibited both PCF cultures and *T. brucei* 24-SMT in vitro. 26-Fluorolanosterol **88** inhibited trypanosome growth with an IC_50_ of about 3 µM, though it was not productively bound in *T. brucei* 24-SMT assays. 26-Fluorolanosterol is a reversible inhibitor of 24-SMT. Conversely, 26-fluorocholesta-5,7,24-trienol **89** is a substrate of 24-SMT, which can be turned over to 24-methylated products or bind irreversibly to the enzyme, with a k_cat_/k_inact_ of 0.26 min^−1^/0.24 min^−1^. Sterol analysis of treated PCF revealed a loss of 24-alkylated sterols as well as a loss of 25(27)-desaturated sterols. Moreover, 26-fluorinated biosynthetic intermediates **90**–**93** downstream from lanosterol (Figure 8b) were detected in 26-fluorolanosterol-treated PCF and human epithelial kidney (HEK) cells. The activity of 26-fluorolanosterol on PCF was attributed to conversion to 26-fluorosterols lacking C4- and C14-methyl groups, capable of irreversibly binding to 24-SMT [67].

The importance of endogenous synthesis of ergostenols in BSF is accentuated by the effectiveness of other reported EBIs [41,43,67,68,69,70,71].

### 3.2. Acanthamoeba *spp*.

**Figure 9:**
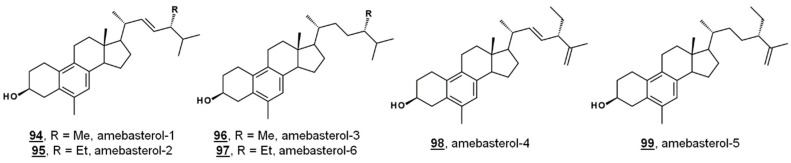
Structures of amebasterols.

Ergosterol is a significant Δ^5^ bulk sterol in amoebae, as is 7-dehydroporiferasterol. Sterols are synthesized *de novo* in amoebae via a biosynthetic pathway involving the protosterol cycloartenol **25**, as in green algae and higher plants. Amoebae also synthesize 19(10→6)-abeo-sterols containing aromatic B rings called the amebasterols [22,36]. Amebasterol-1 **94**, amebasterol-2 **95**, and amebasterol-4 **98** have been described [22]; trace amebasterols-3 **96**, -5 **99**, and -6 **97** have been identified as of late (Figure 9). These compounds can be selectively monitored at UV absorbances of 270 nm [36].

**Figure 10:**
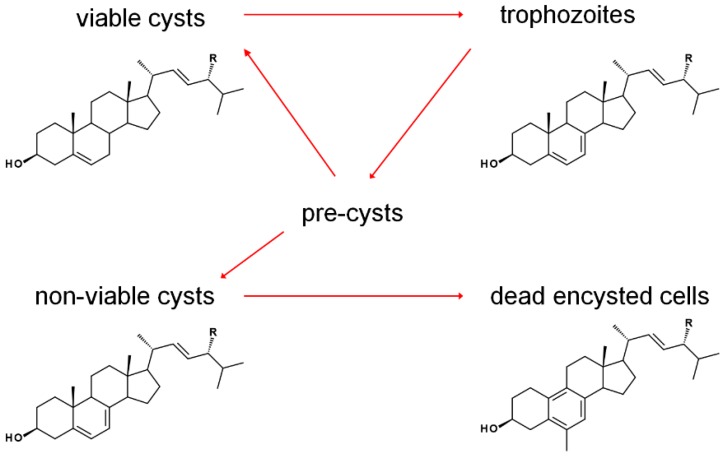
Growth-phase dependence of predominant sterols in *A. castellanii*. R = Me and Et. Adapted from [36].

The sterol profile of was found to be variable as a function of growth and encystment phases. Analysis of the *Acanthamoeba castellanii* sterolome throughout the first week and one month after inoculation revealed a variable composition with changes to cell morphology and viability. At the beginning of the excystment-trophozoite-encystment cycle, in early log phase of growth, an accumulation of protosterol cycloartenol **27** and 24-methylenated cycloartanol **29** was noted. As the cells replicated, trophozoites contained mostly the Δ^5,7^ products ergosterol and 7-dehydroporiferasterol, whereas, in the stationary growth phase, with a mixture of trophozoites and cysts, sterols shifted to the Δ^5^ products brassicasterol and poriferasterol. Supplementation of trophozoite cultures with cholesterol had only a minor stimulation effect on their growth. After one-month incubation, dead cells were mostly comprised of amebasterols, amebasterol-1 **94** and amebasterol-2 **95** (Figure 9). The shift from Δ^5,7^ products in non-viable encysted cells to the amebasterols was attributed to turnover from stress and a sterol composition associated with altered membrane fluidity affording lysis (Figure 10) [36].

Beyond the protosterols, ergosterol/poriferasterol pairs, brassicasterol/poriferasterol pairs, and amebasterol-1/amebasterol-2 pairs, this study identified an additional 13 minor sterols in the metabolome of *A. castellanii*. Labeled experiments with [^2^H_3_]Met elucidated labeling patterns of dideuterated ergosterol and pentadeuterated 7-dehydroporfierasterol, consistent with a Δ^24(28)^ product in its first biomethylation by SMT and a Δ^25(27)^ product in the second biomethylation (*Vs.* Section 2.1) [36]. Labeling outcomes are supported by in vitro mechanisms with recombinant Acanthamoebic SMTs yielding a single Δ^24(28)^ product in the first biomethylation (introduction of C28) [72]. While recombinant SMT yielded both Δ^25(27)^ and Δ^25(27)^ products for the second biomethylation (introduction of C29) [72], the authors concluded the labeling pattern of sterols from [^2^H_3_]Met-fed cultures, indicating that 24(28)-ethyidene sterols are not incorporated into 7-dehydroporiferasterol under physiological conditions [36].

The noted pairs of cycloartenol and 24(28)-methylenecycloartanol (24-H/24-Me), and pairs of ergosterol/poriferasterol, brassicasterol/poriferasterol, and amebasterol-1/amebasterol-2 (each 24-Me/24-Et) in the various portions of the Acanthamoebic life cycle [36], along with product outcomes being largely determined by biomethylation patterns of *A. castellanii* SMTs [36,72], underscores the crucial nature of SMT function in the pathogen. Subtle alterations in substrate selectivity were noted to have a profound impact on the balance of 24-methyl and 24-ethyl sterols [36]. After treatment with the 24-SMT inhibitor 24(*R*,*S*),25-epiminolatnosterol **26** and the azole 14-SDM inhibitor voriconazole **20** (See Figure 2 for structures), and small increase in amounts of cycloartenol and obtusifoliol were noted [72,73]. Upon treatment with EBIs, trohpozoites were stimulated to encyst, while excystment was insensitive to treatment. The correlation between stage-specific sterol compositions and the physiological effects of EBIs provide insight on opportunities for therapeutics (Figure 10). It is imagined that EBIs targeting the enzyme that reduces the Δ^7^ olefin of ergosterol/7-dehydroproferasterol to brassicasterol/poriferasterol could be used to modulate Acanthamoeba growth phases and prevent recurrence of the disease [36].

Azole inhibitors of 14-SDM have been reported to restrict Acanthamoeba growth in the nanomolar to micromolar range [36,37,73,74,75,76], and inhibitors of 24-SMT have been reported with nanomolar activity against Acanthamoeba cultures [36,72]. Treatments of 14-SDM- and 24-SMT-inhibitors in combination led to complete eradication of the amoeba parasite at concentrations as low as their respective IC_50_s [36].

### 3.3. Fungal Sterol Profiles in Drug-Treated Cultures

EBIs are a staple of antimycotic drug discovery [23,24,25]. A general hypothetical biosynthetic pathway, as well as popular block points for EBIs, are presented in Figure 2. Sterolomics can be used to confirm the inhibition of ergosterol biosynthesis upon treatment with new small molecules with antifungal properties.

**Figure 11:**
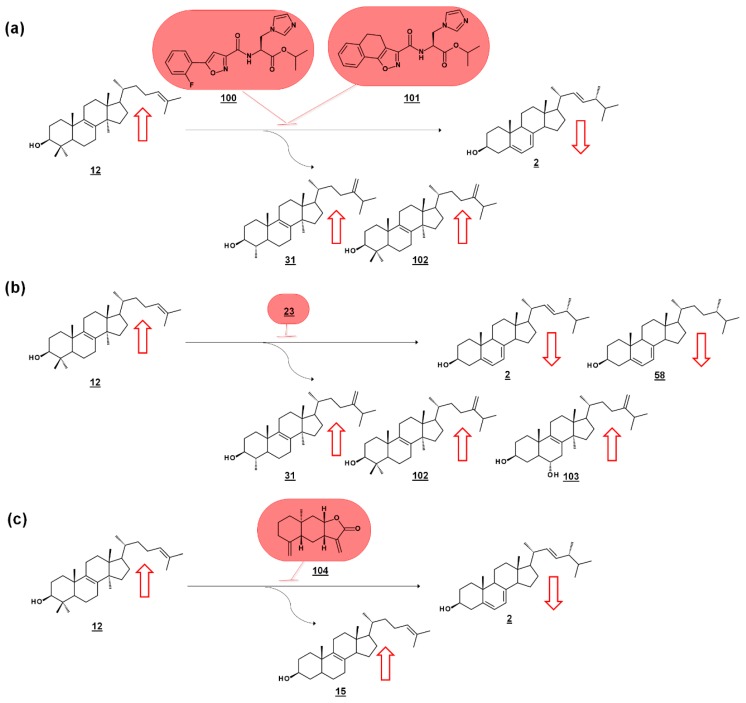
Sterolomic identification of ergosterol biosynthesis inhibitors (EBIs) in fungi. Red arrows signify increase or decrease in sterols within the profile of inhibited cultures relative to non-inhibited cultures. (**a**) Oxazole amidoester-treated cultures of *C. albicans* decrease in ergosterol and increase in lanosterol and by-products obtusifoliol and eburicol, indicating disruption of 14-SDM activity [77,78]. (**b**) Posaconazole-treated cultures of *Rhizopus arrhizus* decrease in ergosterol and ergosta-5,7-dienol and increase in lanosterol, obtusifoliol, and eburicol, and produce toxic 14-methylergosta-8,24(28)-dien-3β,6α-diol [79]. (**c**) *ent*-Isoalantolactone-treated cultures of *C. albicans* decrease in ergosterol and increase in lanosterol and zymosterol, indicating disruption of 24-SMT activity [80].

Series of amidoesters substituted with imidazolylmethyl groups were reported to have bioactivity against opportunistic fungal pathogens *Candida albicans*, *Candida tropicalis*, *Cryptococcus neoformans*, and *Aspergillus fumigatus* [77,78]. Some of these compounds, including **100** [77] and **101** [78] (Figure 11a) displayed better antifungal properties than fluconazole **21** (cf. Figure 2). The sterols of *C. albicans* administered with these compounds were analyzed to confirm a mechanism of disrupting ergosterol biosynthesis. Ergosterol normally comprises of the vast majority of the sterol profile in *C. albicans* (>98%), and treatment with **100** [77] or **101** [78] reduced ergosterol in a dose dependent manner. Dose-dependent increases in lanosterol **12** were noted, as well as increases in 14α-methylsterol by-products eburicol **102** and obtusifoliol **31** (Figure 11a). The increase in lanosterol (substrate for *C. albicans* 14-SDM), the increase in 14-methylsterols, and a commensurate decrease in ergosterol itself, suggested 14-SDM as a target for these molecules [77,78].

Many molds, like clinically relevant Mucorales, methylate the side chain of protosterol lanosterol **12**, before demethylating the sterol nucleus, to produce eburicol **102** as a normal intermediate (Figure 11b). Sterols were examined from six pathogenic molds from the order Mucorales, as well as sterols from cultures treated with the azole drug posaconazole **23** (cf. Figure 2). The untreated molds were reported to contain ergosterol, with prominent composition by ergosta-5,7-dienol **58**. An additional 12 sterols from untreated cultures were reported. *Rhizopus arrhizus* contained 76.3% ergosterol and 10.6% ergosta-5,7-dienol within its sterol fraction. Upon administering sub-lethal concentration of 0.5 µg/mL posaconazole **23**, these percentages were reduced to 58.5% and 5.1%, respectively. Correspondingly, lanosterol and eburicol **102** increased with azole, and other 14-methylsterols were noted. Moreover, non-physiological and toxic 14-methylergosta-8,24(28)-dien-3β,6α-diol **103**, which had only been found prior in azole-dosed yeasts, was detected at 0.7% in treated cells (Figure 11b) [79].

Of a set of sesquiterpenes isolated from Chinese liverwort *Tritomaria quinquedentata (Huds.) Buch.*, 5 exhibited activity against strains of *C. albicans*. The most potent of these compounds, *ent*-isoalantolactone **104** suppressed hyphal formation of the yeast and was further investigated for its antifungal mechanism. An increase in lanosterol **12** and zymosterol **15** was noted in *C. albicans* sterol composition when applied with MIC_80_ concentrations of *ent*-isoalantolactone (Figure 11c). The accumulation of zymosterol connotes inhibition of Erg6p (=24-SMT). Subsequent transcriptional analysis of treated *C. albicans* revealed increased expression of the Erg6 gene 9.3-fold and the Erg11 (=14-SDM) gene 2.7-fold, supporting the sterolomic findings [80].

**Figure 12:**
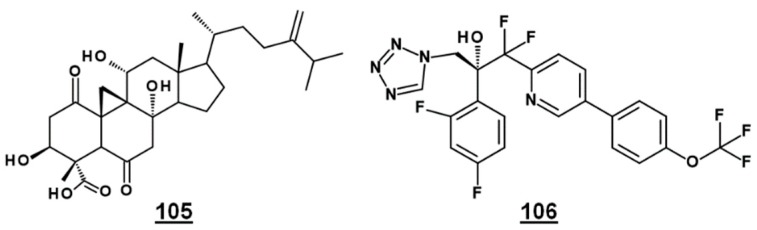
EBIs FR171456 **105** and VT-1129 **106**, confirmed by sterolomic analysis.

Bioactive natural product FR171456 **105** (Figure 12) was shown to inhibit ergosterol biosynthesis of *C. albicans*, by a dose-dependent decrease in labeled zymosterol **15** and ergosterol and increase in labeled lanosterol, upon co-incubation with ^13^C-glucose, ^13^C-acetate. Similarly, fluconazole **21** –treated cultures also decreased in zymosterol and ergosterol [81]. Likewise, investigative drug VT-1129 **106** (Figure 12) caused an increase in lanosterol, eburicol, obtusifoliol, and its 3-ketone analogue, as well as reduction in ergosterol and fungisterol, in *Cryptococcus* sp. [82].

## 4. Bioactive Steroidal Metabolites

Endogenous oxysterols play essential roles in human biology, including signaling, development, and immunology [8,9,10,11]. Similarly, several oxysterols isolated from microbial sources have been reported to exhibit therapeutic properties. Many of these compounds from microbes are oxyphytosterols, i.e., unlike human endogenous sterols, they possess alkyl groups at C24 and therefore do not occur in human biology. Bioactivities include those against cancer cell lines, as well as ligands for nuclear receptors, antioxidants, anti-inflammatory agents, and inhibitors of amyloid-β (Aβ) aggregation.

Minor steroidal metabolites often possess bioactivity against other microbes, like fusidic acid, as discussed above. Study of these natural products can further lead to semi-synthetic analogues for structure-activity relationship studies and improvement of antimicrobial agents. For instance, squalamine **9** (Figure 1c), isolated from dogfish shark, is a steroid with polyamine substitution. The cationic polyamine moiety and its polyvalence have been attributed to much of its antimicrobial and anticancer properties [35], and, as a result, a class of synthetic and semi-synthetic analogues, collectively termed cationic steroid antibiotics, have been developed [35,83,84]. For the purposes of this review, only isolated compounds are discussed, though these compounds can inform synthetic and semisynthetic analogues for increased bioactivity. Likewise, steroidal metabolites with a compromised cyclopentanoperhydrophenanthrene nucleus are omitted here.

### 4.1. Peroxides

**Figure 13:**
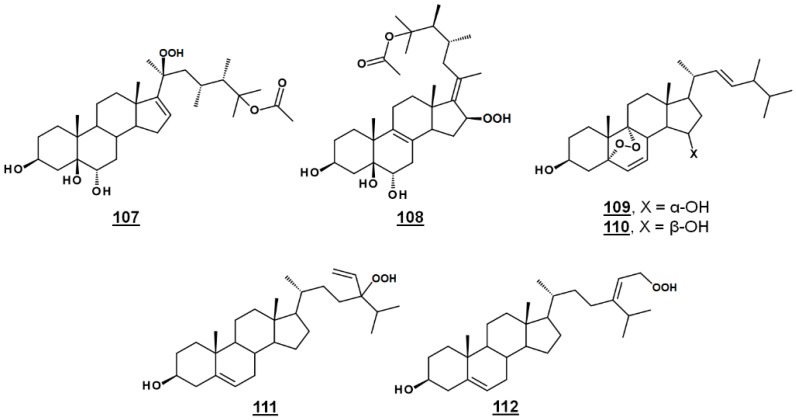
Steryl peroxides discussed in text.

Michosterol A **107** (Figure 13) is a newly described polyoxygenated sterol with a C20 hydroperoxyl group and a C25 acetoxyl group, isolated by the ethyl acetate extract of the soft coral *Lobophytum michaelae*. Michosterol A demonstrated moderate cytotoxic effects against A549 cells, with an IC_50_ of 14.9 µg/mL, and was not cytotoxic (IC_50_s > 20 µg/mL) to DLD-1 and LNCap cell lines. Its anti-inflammatory activity was examined by assaying against superoxide formation in human neutrophils and against elastase release. Michosterol A had IC_50_s of 7.1 µM and 4.5 µM for superoxide anion generation and elastase release, respectively. A second hydroperoxyl polyoxygenated sterol (C15 hydroperoxyl, and Δ^17(20)^), named michosterol B **108** (Figure 13) was discovered in this extract. Michosterol B did not display cytotoxicity against the cell lines tested, but inhibited superoxide anion generation 14.7% and elastase release 31.8% each at 10 µM michosterol B [85].

Nigerasterol A **109** and nigerasterol B **110** (Figure 13) are C15 epimers of 3,15-diols containing a 5,α,9α-peroxide obtained from *Aspergillus niger* MA-132, an endophytic fungus isolated from the mangrove plant *Avicennia marina*. Nigerasterol A and nigerasterol B inhibited cell growth in cancer cell lines HL-60 (IC_50_s 0.3 µM and 1.50 µM, respectively) and A549 (IC_50_s 1.82 µM and 5.41 µM, respectively) [32].

24-Vinyl-24-hydroperoxycholesterol **111** (Figure 13) has been isolated from *Xestponsgia* sp. [33,86]. It had an IC_50_ in an NF-κB-luciferase assay of 31.3 µg/mL [33] and restricted growth of various human cell lines, including A549 (IC_50_ 29.0 µM) and WI-38 (IC_50_ 43.4 µM) [86]. From Xestospongia, the 29-hydroperoxyl derivative **112** (Figure 13) of isofucosterol has also been reported, with broad activity against such targets as NF-κB-luciferase (IC_50_ 12.6 µg/mL), 3-hydroxy-3-methylglutaryl CoA reductase (HMGR)-green fluorescent protein (IC_50_ 3.8 µg/mL) and protein tyrosine phosphatase 1B (IC_50_ 5.8 µg/mL) [33].

### 4.2. Acetates

**Figure 14:**
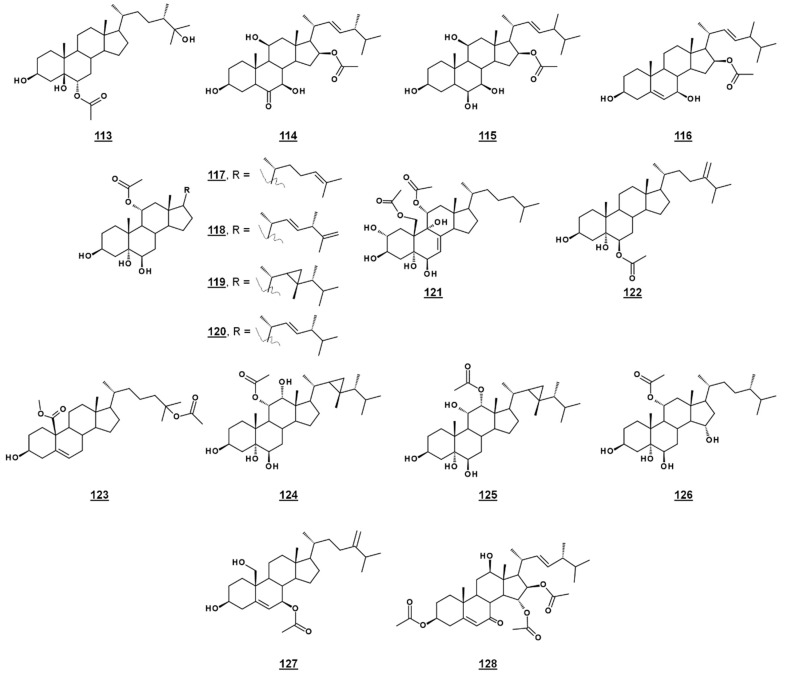
Steryl acetates discussed in text.

A third michosterol, michosterol C **113** (Figure 14), isolated from the soft coral *Lobophytum michaelae* (*Vs.* 4.1. peroxides) lacked a peroxyl moiety, but contained a 6α-acetoxyl group. Michosterol C was not cytotoxic on cell lines tested, but inhibited superoxide anion generation 17.8% at 10 µM and had an IC_50_ for elastase release of 0.9 µM [85].

Anicequol **114** (Figure 14), also known as NGA0187, is a polyhydroxylated ergost-6-one first described in 2002. Originally isolated from the fungi *Penicillium aurantiogriseum Dierckx* TP-F0213 [87] and *Acremonium* sp. TF-0356 [88], Anicequol inhibited anchorage-dependent growth of human colon cancer DLD-1 cells with an IC_50_ of 1.2 µM [87]. Anicequol was found to induce anoikis, or apoptosis by loss of cell adhesion to the extracellular matrix. Induction of anoikis by anicequol, as well as 25-hydroxycholesterol, was additionally found to involve p38 mitogen-activated protein kinase (p38MAPK) and Rho-associated, coiled-coil containing kinase (ROCK), suggesting new therapeutic strategies against cancer [89]. Anicequol has neurotrophic activity and induced significant neurite outgrowth at 30 µg/mL in PC12 cells [88]. Aniceuquol has also been isolated from *Aspergillus terreus* (No. GX7-3B) [90] and *Penicillum chrysogeum* QEN-24S [91], and supplementary activities against α-acetylcholinesterase (AchE) with an IC_50_ of 1.89 µM [90] and against other fungi, with a zone of inhibition (ZOI) of cultures of the pathogen *Alternaria brassicae* of 6 mm compared to 16 mm by amphotericin B [91].

Penicisteroid A **115** (Figure 14) is an analogue of anicequol bearing a 7α-hydroxyl rather than a 7-oxo-group. Extracted from *Penicillium chrysogenum* QEN-24S, an endophytic fungus isolated from a red alga of the genus Laurencia, penicisteroid A exhibited both antimycotic and cytotoxic effects. Against the pathogenic fungi *Aspergillus niger* and *Alternaria brassicae*, penicisteroid A gave ZOIs (20 µg) of 18 mm and 9 mm, respectively, compared to 24 mm and 16 mm for control amphotericin B. Penicisteroid A also inhibited HeLa, SW1990, and NCI-H460 cancer cell lines with IC_50_s of 15 µg/mL, 31 µg/mL, and 40 µg/mL, showing selectivity variable from the anicequol parent compound [91]. Penicisteroid C **116** (Figure 14) also has a C16 acetate, but is less oxygenated than penicisteroid A. It was isolated from a co-cultivation of bacteria *Streptomyces piomogenus* AS63D and fungus *Aspergillus niger* using solid-state fermentation on rice medium. Penicisteroid C displayed selective antimicrobial activity against tested organisms. ZOIs for penicisteroid C were 7 mm, 9 mm, and 10 mm for bacterial cultures *Staphylococcus aureus*, *Bacillus cereus*, and *Bacillus subtilis*, respectively, and were 8 mm and 12 mm for fungal cultures *Candida albicans* and *Saccharomyces cerevisiae*, respectively [92].

A study of the oxysterols from the marine sponge *Haliclona crassiloba* (Figure 14) identified two steryl acetates with antibacterial properties. Newly identified halicrasterol D **120** had minimum inhibition constants (MICs) against tested Gram-positive bacteria ranging from 4 µg/mL against *Enterococcus faecalis* to 128 µg/mL against *S. aureus*. The known diacetate compound **121**, additionally isolated from *H. crassiloba*, had MICs ranging from 8 µg/mL against *S. aureus* to 32 µg/mL *E. faecalis*, in the bacteria tested [93]. A newly identified phytosterol acetate **122** from the soft coral *Sinularia conferta* exhibited low micromolar IC_50_s against cell lines PANC-1 (1.78 µM), A549 (IC_50_ 3.64 µM), and HeLa (19.34 µM) [94]. From Xestospongia, 25-acetoxyl sterol with an oxidized C19 (carboxylate substitution on C10), **123**, was identified and exhibited an IC_50_ against AMP activated protein kinase of 8.5 µg/mL [33]. Acetates **117**–**119** and **124**–**126** (Figure 14) isolated from the coral *Sacrophyton* sp. inhibited Gram-positive and Gram-negative bacteria, with ZOIs ranging from 7.0–14.5 mm for *Escherichia coli* and from 7.5–12.0 mm for *Bacillus megaterium*. They also displayed antifungal properties, inhibiting *Septoria tritci* growth 4.5–10.5 mm [95]. Acetate **127** from the coral *Nephthea erecta* stimulated cytC release and inhibited Akt and mTOR phosphorylation in small cell lung cancer cells, as well as inhibiting tumor growth in the mouse xenograft model [96]. Halymeniaol **128**, an triacetoxyl steroid from the rhodophyte *Halymenia floresii*, was recently reported to have antiplasmodial activity with an IC_50_ of 3.0 µM [97].

### 4.3. Cyclopropanes

**Figure 15:**
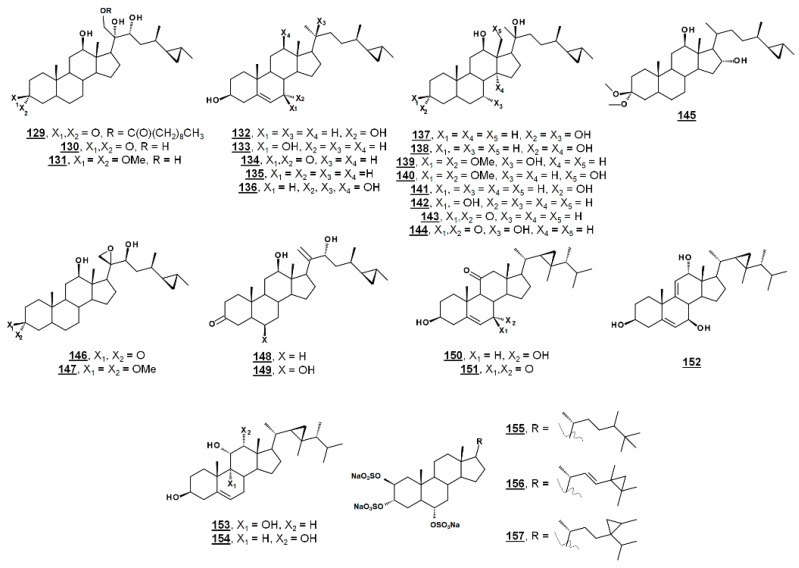
Sterols bearing a 3-membered ring.

From the marine sponge *Xestospongia testudinaria*, oxyphytosterols **129** and **130** (Figure 15), with a side chain cyclized at C26–C27 were recently reported to posess anti-adhesion properties against bacteria *Pseudoalteromonas* spp. and *Polaribacter* sp. New compounds **129** and **133**, as well as known compounds xestokerol A **130**, 7α-hydroxypetrosterol **132**, and aragusterol B **143 ** (Figure 15), had antifouling EC_50_s ranging from 10 to 171 µM. New compound **133** and petrosterol **135** had an EC_50_ > 200 µM [98]. Some of these compounds, other known analogues, and seven new analogues have also been extracted from the marine sponge *Petrosia* (*Strongylophora*) sp. Compounds **130**, **131**, **134**–**141**, and **143**–**147** (Figure 15) displayed micromolar inhibition across various human cancer cell lines tested, with the ketal **139** showing weaker activity [99]. Representatives from this class of steroids from *Xestospongia* spp. tested against human cancer cell line K562 yielded IC_50_s for aragusterol J **149** of IC_50_ 34.31 µM and for aragusterol A **146** of 24.19 µM. Compounds **141**–**143** and **148** had IC_50_s >10 µM [100].

Several oxygenated gorgostenols **150**–**154** (Figure 15), isolated from the soft coral *Klyxum flaccidum* demonstrated selective biological activity. Compounds **150**–**152** and **154** were newly described and named klyflaccisteroids C-E [101] and klyflaccisteroid H [102], respectively. These compounds demonstrated variable inhibition across human cancer cell lines, as well as inhibition of superoxide anion generation and elastase release [101,102]. New analogues of the known trisulfate compound halistanol sulfate **155** bearing cyclopropyl rings on their side chains have been isolated from the marine sponge *Halichondria* sp. Halistanol sulfates I **156** and J **157** had IC_50_ values for sirtuins 1–3 of 45.9, 18.9, and 32.6 µM and 67.9, 21.1, and 37.5 µM, respectively, compared to IC_50_s of the parent structure **105** of 49.1, 19.2, and 21.8 µM [103].

### 4.4. Other Bioactive Steroids

**Figure 16:**
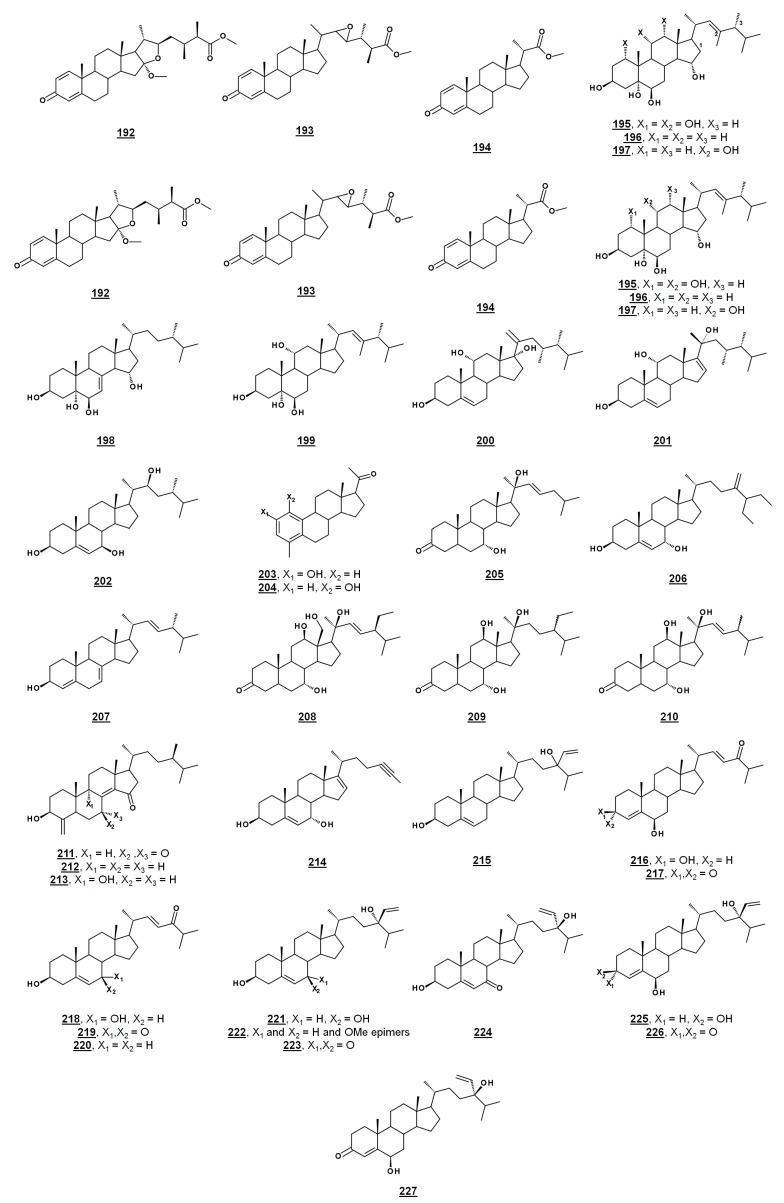
Bioactive sterols and steroids. Activities are given in Table 2.

Several sponge sterols, such as solomonsterol A **158** and B **159**, theonellasterol **160**, conicasterol **161** (Figure 16), and their analogues, can serve as ligands for human nuclear receptors; many of these compounds and their activities have been reviewed [104]. Ganoderic acid A **163** (Figure 16) and related compounds isolated from the higher fungus *Ganoderma* sp. possess broad therapeutic properties, including those of anti-tumor and anti-inflammation [105]. Additional recently reported bioactivities of sterols from microorganisms and algae are presented in Table 2 and Figure 16.

## 5. Conclusions

Metabolomics of sterols in microorganisms have provided insight into the biology of microorganisms. Steroidal chemotaxonomy can be used to elucidate phylogenic relationships and steroidal biomarkers can be used to monitor microbial growth and biomass production. Sterolomics additionally plays an influential role in drug discovery, through validation of drug targets, by confirmation of small molecule mechanisms, and by biological testing of microbial metabolites. The sterolome of microbiota can inform chemical biology, evolutionary traits, ecology, and pharmacology.

## Figures and Tables

**Table 1:** Recently reported sterol profiles from algae across classes.

Algal Organism	Major Sterols ^1^ (>40%)	Semi-Major Sterols ^1^ (>20%)	Minor Sterols ^1^ (<20%)	Reference ^2^
Ulvophyceae				
*Acetabularia caliculus*	(3/37)		(36/38), (39/40), (41/42), 44, (48/49)	[52]
*Acicularia schenckii*	(3/37)		(36/38), (39/40), (41/42), 44	[52]
Trebouxiophyceae				
*Chlorella vulgaris*	2	52	56, 58, 64, 62, 66	[53]
*Chlorella luteoviridis*	40	38	37, 42, 52	[53]
Eustigmatophyceae				
*Nannochloropsis limnetica*	1		44, (3/37), 45, (67/68)	[53]
Bacillariophyceae (diatoms)				
*Stephanodiscus hantzschii*	45		48, 17, 57, 46	[53]
*Gomphonema parvulum*	41		(69/2), (59/60), (36,38)	[53]
*Cyclotella meneghiniana*	45		38, 43, 48	[53]
Raphidophyceae				
*Chloromorum toxicum*	40		37, 1, 42, 38, 48	[54]
*Chattonella marina*	3		1, 63	[54]
*Heterosigma akashiwo*	37		38	[54]
Dictyochophyceae				
*Verrucophora farcimen*	70			[54]
Chlorophyceae (see also Figure 3)				
*Scenedesmus obliquus*	54	52	50, 62, 60	[53]
*Monoraphidium mintutum*	50	52	65, 54, 62, 60, 64	[53]
Cryptophyceae				
*Cryptomonas* sp.	39, 41			[53]
*Rhodomonas* sp.	41			[55]

^1^ Major, semi-major, and minor components of algal sterols as a percentage of total sterol. Numbers refer to structures in Figure 4 and earlier. Parenthetical pairs are provided for epimers, for which C24 stereochemistry was not reported. ^2^ Reference.

**Table 2:** Recently reported biological activities from microbial steroids.

No.^1^	Microbial Source	Biological Target ^2^	Biological Activity	Reference
Fungi				
**163**	*Nigrospora sphaerica*	Cryptococcus neoformans	IC_50_ 14.81 µg/mL	[106]
**164**	*Gymnoascus reessii*	NCI-H187	IC_50_ 16.3 µg/mL	[34]
		*Plasmodium falciparum*	IC_50_ 3.3 µg/mL	[34]
**165**	*Gymnoascus reessii*	NCI-H187	IC_50_ 47.9 µg/mL	[34]
		*Plasmodium falciparum*	IC_50_ 4.5 µg/mL	[34]
**166**	*Gymnoascus reessii*	NCI-H187	IC_50_ 1.9 µg/mL	[34]
		*Plasmodium falciparum*	IC_50_ 3.4 µg/mL	[34]
**167**	*Gymnoascus reessii*	NCI-H187	IC_50_ 12.5 µg/mL	[34]
		*Plasmodium falciparum*	IC_50_ 3.4 µg/mL	[34]
**168**	*Aspergillus* sp.	*Balanus amphitrite* biofouling	EC_50_ 18.40 µg/mL	[107]
**169**	*Nodulisporium* sp.	Aβ_42_ aggregation	IC_50_ 10.1 µM	[108]
**170**	*Nodulisporium* sp.	Aβ_42_ aggregation	54.6% relative inhibitory activity at 100 µM	[108]
**171**	*Nodulisporium* sp.	Aβ_42_ aggregation	IC_50_ 1.2 µM	[108]
**172**	*Nodulisporium* sp.	Aβ_42_ aggregation	IC_50_ 43.5 µM	[108]
**173**	*Dichotomomyces cejpii*	Aβ_42_ aggregation	pretreatment with 10 µM reduced production of Aβ peptides to 3.8-fold increase with 100 µM Aftin-5 compared to 9.4-fold increase with only Aftin-5 and no inhibitor	[109]
Coral				
**174**	*Sinularia nanolobata*	HL-60	IC_50_ 33.53 µM	[110]
		HepG2	IC_50_ 64.35 µM	[110]
**175**	*Sinularia microspiculata*	HL-60	IC_50_ 82.80 µM	[111]
		SK-Mel2	IC_50_ 72.32 µM	[111]
**176**	*Sinularia leptoclados*	HL-60	IC_50_ 13.45 µM	[112]
		SW480	IC_50_ 14.42 µM	[112]
		LNCaP	IC_50_ 17.13 µM	[112]
		MCF-7	IC_50_ 17.29 µM	[112]
**177**	*Sinularia leptoclados*	HL-60	IC_50_ 20.53 µM	[112]
		SW480	IC_50_ 26.61 µM	[112]
		KB	IC_50_ 32.86 µM	[112]
**178**	*Sinularia conferta*	A549	IC_50_ 78.73 µM	[94]
		HeLa	IC_50_ 30.5 µM	[94]
		PANC-1	IC_50_ 9.35 µM	[94]
**179**	*Sinularia conferta*	A549	IC_50_ 27.12 µM	[94]
		HeLa	IC_50_ 24.64 µM	[94]
		PANC-1	IC_50_ 20.51 µM	[94]
**180**	*Sinularia brassica*	PANC-1	IC_50_ 15.24 µM	[113]
		A549	IC_50_ 39.36 µM	[113]
**181**	*Sinularia brassica*	PANC-1	IC_50_ 22.47 µM	[113]
		A549	IC_50_ 41.20 µM	[113]
**182**	*Sinularia brassica*	A549	IC_50_ 47.31 µM	[113]
**183**	*Sinularia brassica*	PANC-1	IC_50_ 15.39 µM	[113]
		A549	IC_50_ 47.46 µM	[113]
**184**	*Sinularia brassica*	PANC-1	IC_50_ 38.12 µM	[113]
		A549	IC_50_ 23.73 µM	[113]
**185**	*Sinularia brassica*	A549	IC_50_ 92.53 µM	[113]
	*Sacrcophyton* sp.	*E. coli*	0.05 mg ZOI ^3^ 10.0 mm	[95]
		*S. tritici*	0.05 mg ZOI 7.5 mm	[95]
**187**	*Sinularia microspiculata*	HL-60	IC_50_ 89.02 µM	[111]
**188**	*Sinularia* sp.	HL-60	IC_50_ 1.79 µM	[114]
**189**	*Sinularia* sp.	HL-60	IC_50_ 4.03 µM	[114]
**190**	*Sinularia* sp.	HL-60	IC_50_ 0.69 µM	[114]
**191**	*Sinularia brassica*	P388D1	IC_50_ 37.2 µM	[115]
		MOLT-4	IC_50_ 37.8 µM	[115]
**192**	*Sinularia brassica*	P388D1	IC_50_ 9.7 µM	[115]
		MOLT-4	IC_50_ 6.0 µM	[115]
**193**	*Sinularia brassica*	P388D1	IC_50_ 5.7 µM	[115]
		MOLT-4	IC_50_ 5.3 µM	[115]
**194**	*Sinularia brassica*	P388D1	IC_50_ 24.4 µM	[115]
		MOLT-4	IC_50_ 31.2 µM	[115]
**186**	*Sacrcophyton* sp.	*E. coli*	0.05 mg ZOI 5.0 mm	[95]
		*S. tritici*	0.05 mg ZOI 7.0 mm	[95]
**195**	*Sacrcophyton* sp.	*E. coli*	0.05 mg ZOI 7.5 mm	[95]
		*S. tritici*	0.05 mg ZOI 10.5 mm	[95]
**196**	*Sacrcophyton* sp.	*E. coli*	0.05 mg ZOI 4.5 mm	[95]
		*S. tritici*	0.05 mg ZOI 6.5 mm	[95]
**197**	*Sacrcophyton* sp.	*E. coli*	0.05 mg ZOI 6.0 mm	[95]
		*S. tritici*	0.05 mg ZOI 4.5 mm	[95]
**198**	*Sacrcophyton* sp.	*E. coli*	0.05 mg ZOI 6.0 mm	[95]
		*S. tritici*	0.05 mg ZOI 6.0 mm	[95]
**199**	*Sacrcophyton* sp.	*E. coli*	0.05 mg ZOI 6.0 mm	[95]
		*S. tritici*	0.05 mg ZOI 9.0 mm	[95]
**200**	*Klyxum flaccidum*	A549	ED_50_ 7.7 µg/mL	[101]
**201**	*Klyxum flaccidum*	K562	IC_50_ 12.7 µM	[102]
		elastase release	IC_50_ 4.40 µM	[102]
**202**	*Klyxum flaccidum*	P388	IC_50_ 31.8 µM	[116]
		elastase release	IC_50_ 5.84 µM	[116]
**203**	*Subergorgia suberosa*	Influenza virus strain A/WSN/33 (H1N1)	IC_50_ 37.73 µM	[117]
**204**	*Subergorgia suberosa*	Influenza virus strain A/WSN/33 (H1N1)	IC_50_ 50.95 µM	[117]
Sponges				
**205**	*Petrosia* sp.	MOLT-3	IC_50_ 36.57 µM	[99]
		A549	IC_50_ 54.26 µM	[99]
**206**	*Xestospongia testudinaria*	MCF-7	IC_50_ 55.8 µM	[86]
		A549	IC_50_ 63.1 µM	[86]
**207**	*Xestospongia testudinaria*	PTP1B ^4^	IC_50_ 4.27 µM	[118]
**208**	*Xestospongia* sp.	K562	IC_50_ 18.32 µM	[100]
**209**	Xestospongia sp.	K562	25.73% inhibition at 10 µM	[100]
**210**	Xestospongia sp.	K562	41.32% inhibition at 10 µM	[100]
**158**	*Theonella swinhoei*	arthritis	30% reduction in clinical arthritis scores in mice treated with 10 mg/kg	[119]
**211**	*Theonella swinhoei*	U937	IC_50_ 8.8 µM	[120]
		PC-9	IC_50_ 7.7 µM	[120]
**212**	*Theonella swinhoei*	U937	IC_50_ 2.0 µM	[120]
		PC-9	IC_50_ 9.7 µM	[120]
**213**	*Theonella swinhoei*	U937	IC_50_ 3.2 µM	[120]
		PC-9	IC_50_ 1.6 µM	[120]
**214**	*Callyspongia* aff. *implexa*	*Chlamydia trachomatis*	IC_50_ 2.3 µM	[121]
Brown Algae				
**44**	*Sargassum linearifolium*	*Plasmodium falciparum*	IC_50_ 7.48 µg/mL	[122]
**215**	*Sargassum muticum*	obesity	decreased lipid accumulation and dose-dependent suppression of PPARγ ^5^	[123]
	*Sargassum fusiform*	depression	32.67/53.60 and 32.06/50.83 percentage decrease in immobility duration for forced swimmin and tail suspension test in the mouse model at 10 mg/kg/30 mg/kg	[124]
**216**	*Dictyopteris undulata* Holmes	PTP1B ^4^	IC_50_ 7.92 µM	[125]
**217**	*Dictyopteris undulata* Holmes	PTP1B ^4^	IC_50_ 7.78 µM	[125]
**218**	*Dictyopteris undulata* Holmes	PTP1B ^4^	IC_50_ 3.03 µM	[125]
**219**	*Dictyopteris undulata* Holmes	PTP1B ^4^	IC_50_ 3.72 µM	[125]
**220**	*Dictyopteris undulata* Holmes	PTP1B ^4^	IC_50_ 15.01µM	[125]
**221**	*Dictyopteris undulata* Holmes	PTP1B ^4^	IC_50_ 35.01 µM	[126]
**222**	*Dictyopteris undulata* Holmes	PTP1B ^4^	IC_50_ 1.88 µM	[126]
		HL-60	IC_50_ 2.08 µM	[126]
**223**	*Dictyopteris undulata* Holmes	HL-60	IC_50_ 2.45 µM	[126]
**224**	*Dictyopteris undulata* Holmes	PTP1B ^4^	IC_50_ 38.15 µM	[126]
		HL-60	IC_50_ 2.70 µM	[126]
**225**	*Dictyopteris undulata* Holmes	PTP1B ^4^	IC_50_ 48.21 µM	[126]
		HL-60	IC_50_ 1.02 µM	[126]
**226**	*Dictyopteris undulata* Holmes	PTP1B ^4^	IC_50_ 3.47 µM	[126]
		HL-60	IC_50_ 1.26 µM	[126]
**227**	*Dictyopteris undulata* Holmes	PTP1B ^4^	IC_50_ 16.03 µM	[126]
		HL-60	IC_50_ 1.17 µM	[126]

^1^ Compound number. Structures are given in Figure 16. ^2^ Cancer cell lines include A549, lung adenocarcinoma; HeLa, cervical adenocarcinoma; HepG2, hepatocellular carcinoma; HL-60, promyelocytic leukemia; KB, epidermoid carcinoma; K562, bone marrow myelogenous leukemia; LNCaP, prostate cancer; MCF-7, breast adenocarcinoma; MOLT-4, lymphoblastic leukemia; NCI-H187, lung carcinoma; PANC-1, pancreatic epithelioid carcinoma; PC-9, lung adenocarcinoma; P388, murine leukemia; P388D1, lymphoma; SK-Mel2, melanoma; SW480, colorectal adenocarcinoma; U937, histiocytic lymphoma. ^3^ ZOI, zone of inhibition. ^4^ PTP1B, protein tyrosine phosphatase 1B. ^5^ PPARγ, peroxisome proliferator-activated receptor γ.

## Abbreviations

[^2^H_3_]AdoMet	methyl-trideuterated *S*-adenosylmethionine
[^2^H_3_]Met	methyl-trideuterated methionine
8(7)-SI	sterol C8(7)-isomerase
14-SDM	sterol C14-demethylase
14-SR	sterol C14-sterol reductase
24-SMT	sterol C24-methyltransferase
Aβ	amyloid-β
AchE	α-acetylcholinesterase
BSF	bloodstream form
EBIs	ergosterol biosynthesis inhibitors
FF-MAS	follicular fluid meiosis-activating sterol
HEK	human epithelial kidney
HMGR	3-hydroxy-3-methylglutaryl CoA reductase
MASs	meiosis-activating sterols
T-MAS	testicular meiosis-activating sterol
OSC	oxidosqualene cyclase
p38MAPK	p38 mitogen-activated protein kinase
PCF	procyclic form
PPARγ	peroxisome proliferator-activated receptor γ
PTP1B	protein tyrosine phosphatase 1B
ROCK	Rho-associated coiled-coil containing kinase
SqE	squalene epoxidase; TMS, trimethylsilyl(ated)
ZOI	zone of inhibition

## Appendix A

Table A1 gives the systematic names of all steroids discussed in the text.

**Table A1:** Trivial and systematic names of sterols depicted in figures and discussed in text.

No.^1^	Trivial Name, If Applicable (Secondary Trivial Name, If Applicable)	Systematic Name ^2^ (Systematic Name Relative to 5α-Cholestane)	PubChem CID
**1**	cholesterol	cholest-5-en-3β-ol	5997
**2**	ergosterol	ergosta-5,7,22*E*-trien-3β-ol (24β-methylcholesta-5,7,22*E*-trien-3β-ol)	444679
**3**	sitosterol	stigmast-5-en-3β-ol (24α-methylcholest-5-en-3β-ol)	222284
**4**	FF-MAS	4α,4β-dimethylcholesta-8,14,24-trien-3β-ol	443212
**5**	T-MAS	4α,4β-dimethylcholesta-8,24-dien-3β-ol	50990081
**6**	25-hydroxycholesterol	cholest-5-en-3β,25-diol	65094
**8**	ergosterol peroxide	5α,8α-epidioxyergosta-6,22*E*-dien-3β-ol (24β-methyl-5α,8α-epidioxycholesta-6,22*E*-dien-3β-ol)	5351516
**9**	squalamine	3β-[3-(4-aminobutyl)amino]propyl-7α-hydroxycholestan-24β-hydrosulfate	72495
**12**	lanosterol	lanosta-8,24-dien-3β-ol (4α,4β,14α-trimethylcholesta-8,24-dien-3β-ol)	246983
**13**	31-norlanosterol	4α,14α-dimethylcholesta-8,24-dien-3β-ol	15101557
**14**	zymosterone	cholesta-8,24-dien-3-one	22298942
**15**	zymosterol	cholesta-8,24-dien-3β-ol	92746
**16**	fecosterol	ergosta-8,24(28)-dien-3β-ol (24-methylideneholest-8-en-3β-ol)	440371
**17**	episterol	ergosta-7,24(28)-dien-3β-ol (24-methylideneholest-7-en-3β-ol)	5283662
**18**		ergosta-5,7,22*E*,24(28)-tetraen-3β-ol (24-methylideneholesta-5,7,22*E*-trien-3β-ol)	11090531
**25**	25-azalanosterol	25-azalanost-8(9)-en-**3**β-ol 4α,4β,14α-trimethyl-25-azacholest-8-en-3β-ol	66746490
**26**	24(R,S),25-epiminolanosterol	24(*R*,*S*),25-epiminolanost8(9)-en-**3**β-ol (4α,4β,14α-trimethyl-24,25-azanetriylcholest-8-en-3β-ol)	163740
**27**	cycloartenol	cycloart-24(25)-en-**3**β-ol (4α,4β,14α-trimethyl-9β,19-cyclocholest-24-en-3β-ol)	92110
**28**	cyclolaudenol	24β-methylcycloart-25(27)-en-**3**β-ol (4α,4β,14α,24β-tetramethyl-9β,19-cyclocholest-25(27)-en-3β-ol)	101729
**29**	24-methylenecycloartanol	24-methylidenecycloartan-**3**β-ol (4α,4β,14α-trimethyl-24-methylidene-9β,19-cyclocholestan-3β-ol)	94204
**30**		ergosta-8,25(27)-dien-**3**β-ol (24β-methylcholest-8,25(27)-dien-3β-ol)	102515129
**31**	obtusifoliol	4α,14α-dimethylergosta-8,24(28)-dien-3β-ol (4α,14α-dimethyl-24-methylideneholest-8-en-3β-ol)	65252
**32**	24(28)-methylenelophenol	4α-methylergosta-7,24(28)-dien-3β-ol (4α-methyl-24-methylideneholest-7-en-3β-ol)	5283640
**33**	chlamysterol	4α,14α-dimethylporiferasta-8,25(27)-dien-3β-ol (24β-ethyl-4α,14α-dimethyl-cholesta-8,25(27)-dien-3β-ol)	90657605
**34**	24(28)*Z*-ethylidene lophenol (citrostadienol)	4α-methylstigmasta-7,24(28)*Z*-dien-3β-ol (4α-methyl-24*Z*-ethylideneholest-7-en-3β-ol)	9548595
**35**	7-dehydroporiferasterol	poriferasta-5,7,22*E*-trien-**3**β-ol (24β-ethylcholesta-5,7,22*E*-trien-3β-ol)	20843308
**36**	campesterol	campest-5-en-**3**β-ol (24α-methylcholest-5-en-3β-ol)	173183
**37**	clionosterol (22-dihydroporiferasterol)	poriferast-5-en-**3**β-ol (24β-ethylcholest-5-en-3β-ol)	457801
**38**	22-dihydrobrassicasterol	ergost-5-en-**3**β-ol (24β-methylcholesta-5-en-3β-ol)	312822
**39**	stigmasterol	stigmasta-5,22*E*-dien-**3**β-ol (24α-ethylcholesta-5,22*E*-dien-3β-ol)	5280794
**40**	poriferasterol	poriferasta-5,22*E*-dien-**3**β-ol (24β-ethylcholesta-5,22*E*-dien-3β-ol)	5281330
**41**	crinosterol (epibrassiasterol)	campesta-5,22*E*-dien-**3**β-ol (24α-methylcholesta-5,22*E*-dien-3β-ol)	5283660
**42**	brassicasterol	ergosta-5,22*E*-**3**β-ol (24β-methylcholesta-5,22*E*-dien-3β-ol)	5281327
**43**	fucosterol	stigmasta-5,24(28)*E*-dien-**3**β-ol (24*E*-ethylideneholest-7-en-3β-ol)	5281328
**44**	isofucosterol	stigmasta-5,24(28)*Z*-dien-**3**β-ol (24*Z*-ethylideneholest-5-en-3β-ol)	5281326
**45**	24(28)-methylenecholesterol (chalinasterol)	ergosta-5,24(28)-dien-**3**β-ol (24-methylideneholest-5-en-3β-ol)	92113
**46**	24-ethyldesmosterol	stigmasta-5,24(25)-dien-**3**β-ol (24-ethylcholesta-5,24(25)-dien-**3**β-ol)	22848721
**47**	24-methyldesmosterol	ergosta-5,24(25)-dien-**3**β-ol (24-methylcholesta-5,24(25)-dien-**3**β-ol)	193567
**48**	desmosterol	cholesta-5,24-dien-3β-ol	439577
**49**	schottenol	stigmast-7-en-**3**β-ol (24α-ethylcholest-7-en-3β-ol)	441837
**50**	22-dihydrochondrillasterol	poriferast-7-en-**3**β-ol (24β-ethylcholest-7-en-3β-ol)	5283639
**51**	epifungisterol (22-dihydrostellasterol)	campest-7-en-**3**β-ol (24α-methylcholest-7-en-3β-ol)	90889779
**52**	fungisterol	ergost-7-en-**3**β-ol (24β-methylcholest-7-en-3β-ol)	5283646
**53**		stigmasta-7,22*E*-dien-**3**β-ol (24α-ethylcholesta-7,22*E*-dien-3β-ol)	125122456
**54**	chondrillasterol	poriferasta-7,22*E*-dien-**3**β-ol (24β-ethylcholesta-7,22*E*-dien-3β-ol)	5283663
**55**	stellasterol	campesta-7,22*E*-dien-**3**β-ol (24α-methylcholest-7,22*E*-dien-3β-ol)	5283669
**56**	5-dihydroergosterol	ergosta-7,22*E*-dien-**3**β-ol (24β-methylcholest-7,22*E*-dien-3β-ol)	13889661
**57**	24-dehydrolathosterol	cholesta-7,24-dien-**3**β-ol	5459827
**58**	22-dihydroergosterol	ergosta-5,7-dien-**3**β-ol (24β-methylcholest-5,7-dien-3β-ol)	5326970
**59**		stigmast-8-en-**3**β-ol (24α-ethylcholest-8-en-3β-ol)	23424905
**60**		poriferast-8-en-**3**β-ol (24β-ethylcholest-8-en-3β-ol)	101826503
**61**		campest-8-en-**3**β-ol (24α-methylcholest-8-en-3β-ol)	-
**62**		ergost-8-en-**3**β-ol (24β-methylcholest-8-en-3β-ol)	60077053
**63**	cholest-8-enol (24-dihydrozymosterol)	cholest-8-en-3β-ol	101770
**64**		ergosta-7,25(27)-dien-3β-ol (24β-methylcholesta-7,25(27)-dien-3β-ol)	60077052
**65**		poriferasta-7,25(27)-dien-3β-ol (24β-ethylcholesta-7,25(27)-dien-3β-ol)	5283655
**66**	lichesterol	ergosta-5,8,22*E*-trien-3β-ol (24β-methylcholesta-5,8,22*E*-trien-3β-ol)	5281329
**67**	clerosterol	poriferasta-5,25(27)-dien-3β-ol (24β-ethylcholesta-5,25(27)-dien-3β-ol)	5283638
**68**	epiclerosterol	stigmasta-5,25(27)-dien-3β-ol (24α-ethylcholesta-5,25(27)-dien-3β-ol)	185472
**69**	5-dehydrostellasterol (epiergosterol)	campesta-5,7,22*E*-trien-3β-ol (24α-methylcholesta-5,7,22*E*-trien-3β-ol)	124427258
**70**	occelasterol	27-norcholesta-5,22*E*-dien-3β-ol	15481847
**71**	lathosterol	cholest-7-en-3β-ol	65728
**72**	7-dehydrocholesterol (provitamin D3)	cholesta-5,7-dien-3β-ol	439423
**73**	fucosteryl epoxide	24,28-epoxyergost-5-en-3β-ol 24-(3-methyloxiran-2-yl)-cholest-5-en-3β-ol	3082427
**74**	dinosterol	4α,23-dimethylergost-22*E*-en-3β-ol (4α,23,24β-trimethylcholest-22*E*-en-3β-ol)	44263330
**75**		24*Z*-propylidenecholesterol	6443745
**76**	dihydrodinosterol	(23*R*)-4α,23-dimethylergostan-3β-ol ((23*R*)-4α,23,24β-trimethylcholestan-3β-ol)	133309
**77**	amphisterol	4α-methylergosta-8(14),24(28)-dien-3β-ol (4α-methyl-24-methylidenechoest-8(14)-en-3β-ol)	60077061
**78**	4α-methylergosterol	4α-methylergosta-5,7,22E-trien-3β-ol (4α,24β-dimethylcholesta-5,7,22E-trien-3β-ol)	-
**79**	4α-methylgorgosterol	4α-methylgorgost-5-en-3β-ol ((22*R*,23*R*)-4α,23,24β-trimethyl-22,23-methanocholest-5-en-3β-ol)	-
**80**	gorgosterol	gorgost-5-en-3β-ol ((22*R*,23*R*)-23,24β-dimethyl-22,23-methanocholest-5-en-3β-ol)	52931413
**81**	cholestanol	(5α) cholestan-3β-ol	6710664
**82**	cholesta-5,7,24-trienol 7-dehydrodesmosterol	cholesta-5,7,24-trien-3β-ol	440558
**83**		ergosta-5,7,24(28)-trien-3β-ol (24β-methylcholesta-5,7,24(28)-trien-3β-ol)	10894570
**84**		ergosta-5,7,24(25)-trien-3β-ol (24β-methylcholesta-5,7,24(25)-trien-3β-ol)	58104987
**85**		ergosta-5,7,25(27)-trien-3β-ol (24β-methylcholesta-5,7,25(27)-trien-3β-ol)	101600336
**86**		24,24-dimethylcholesta-5,7,25(27)-trien-3β-ol	-
**87**	protothecasterol	ergosta-5,7,22*E*,25(27)-tetraen-3β-ol (24β-methylcholesta-5,7,22*E*,25(27)-tetraen-3β-ol)	101600338
**88**	26-fluorolanosterol	26-fluorolanosta-8,24-dien-3β-ol (26-fluoro-4α,4β,14α-trimethylcholesta-8,24-dien-3β-ol)	-
**89**		26-fluorocholesta-5,7,24-trien-3β-ol	-
**90**		26-fluoro-4α,4β-dimethylcholesta-8,24-dien-3β-ol	-
**91**		26-fluoro-4α-methylcholesta-8,24-dien-3β-ol	-
**92**		26-fluorocholesta-8,24-dien-3β-ol	-
**93**		26-fluoroergosta-8,25(27)-dien-3β-ol 26-fluoro-24β-methylcholesta-8,25(27)-dien-3β-ol	-
**94**	amebasterol-1	19(10→6)-abeo-ergosta-5,7,9,22*E*-tetraen-3β-ol (24β-methyl-19(10→6)-abeo-cholesta-5,7,9,22*E*-tetraen-3β-ol)	11596359
**95**	amebasterol-2	19(10→6)-abeo-poriferasta-5,7,9,22*E*-tetraen-3β-ol (24β-ethyl-19(10→6)-abeo-cholesta-5,7,9,22*E*-tetraen-3β-ol)	-
**96**	amebasterol-3	19(10→6)-abeo-ergosta-5,7,9-trien-3β-ol (24β-methyl-19(10→6)-abeo-ergosta-5,7,9-trien-3β-ol)	-
**97**	amebasterol-4	19(10→6)-abeo-poriferasta-5,7,9,22*E*,25-pentaen-3β-ol (24β-ethyl-19(10→6)-abeo-cholesta-5,7,9,22*E*,25-pentaen-3β-ol)	-
**98**	amebasterol-5	19(10→6)-abeo-poriferasta-5,7,9,25(27)-tetraen-3β-ol (24β-ethyl-19(10→6)-abeo-cholesta-5,7,9,25(27)-tetraen-3β-ol)	-
**99**	amebasterol-6	19(10→6)-abeo-poriferasta-5,7,9-trien-3β-ol (24β-ethyl-19(10→6)-abeo-poriferasta-5,7,9-trien-3β-ol)	-
**102**	eburicol	24-methylidenelanost-8-en-3β-ol 4α,4β,14α-trimethyl-24-methylidenecholest-8-en-3β-ol	9803310
**103**		14-methylergosta-8,24(28)-dien-3β,6α-diol 14,24β-dimethyl-24-methylidenecholest-8-en--3β,6α-diol	148910
**105**	FR171456	24-methylidene-3β,8α,11α-trihydroxy-1,6-dioxocycloartan-30-oic acid (4α,14α-dimethyl-24-methylidene-9β,19-cyclo-3β,8α,11α-trihydroxy-1,6-dioxocholestan-4β-carboxylic acid)	-
**107**	michosterol A	(20*S*,23*R*)-23-methyl-20-hydroperoxy-25-acetoxyergost-16-en-3β,5β,6α-triol ((20*S*,23*R*)-23,24β-dimethyl-20-hydroperoxy-25-acetoxycholest-16-en-3β,5β,6α-triol)	-
**108**	michosterol B	(17*E*,23*R*)-23-methyl-16-hydroperoxy-25-acetoxyergost-17-en-3β,5β,6α-triol ((17*E*,23*R*)-23,24β-di-methyl-16-hydroperoxy-25-acetoxycholest-17-en-3β,5β,6α-triol)	-
**109**	nigerasterol A	5α,9α-epidioxyergosta-6,8(14),22*E*-trien-3β,15α-diol (24β-methyl-5α,9α-epidioxycholesta-6,8(14),22*E*-trien-3β,15α-diol)	-
**110**	nigerasterol B	5α,9α-epidioxyergosta-6,8(14),22*E*-trien-3β,15β-diol (24β-methyl-5α,9α-epidioxycholesta-6,8(14),22*E*-trien-3β,15β-diol)	-
**111**		24-ethenyl-24-hydroperoxycholest-5-en-3β-ol	10411225
**112**	29-hydroperoxyisofucosterol	(24Z)-29-hydroperoxystigmasta-5,24(28)-dien-3β-ol 24*Z*-(2-hydroperoxyethylidnene)cholest-5-en-3β-ol	46224335
**113**	michosterol C	6α-acetoxyergostan-3β,5β,25-triol (24β-methyl-6α-acetoxycholestan-3β,5β,25-triol)	-
**114**	anicequol (NGA0187)	16β-acetoxy-3β,7β,11β-trihydroxyergost-22*E*-en-6-one (24β-methyl-16β-acetoxy-3β,7β,11β-trihydroxycholest-22*E*-en-6-one)	10413810
**115**	penicisteroid A	24-methyl-16β-acetoxycholest-22*E*-en-3β,6β,7β,11β-tetrol	-
**116**	penicisteroid C	24-methyl-16β-acetoxycholesta-5,22*E*-dien-3β,6β-diol	-
**117**		11α-acetoxycholest-24-en-3β,5α,6β-triol	-
**118**		11α-acetoxyergosta-22*E*,25-dien-3β,5α,6β-triol 24β-methyl-11α-acetoxycholesta-22*E*,25-dien-3β,5α,6β-triol	-
**119**		11α-acetoxygorgostan-3β,5α,6β-triol ((22*R*,23*R*)-23,24β-dimethyl-22,23-methano-11α-acetoxycholestan-3β,5α,6β-triol)	54769262
**120**	halicrasterol D	11α-acetoxyergost-22*E*-en-3β,5α,6β-triol 24β-methyl-11α-acetoxycholest-22*E*-en-3β,5α,6β-triol	-
**121**		11α,19-diacetoxycholest-7-en-2α,3β,5α,6β,9α-pentol	-
**122**		6β-acetoxyergost-24(28)-en-3β,5α-diol 24-methylidene-6β-acetoxycholestan -3β,5α-diol	101687891
**123**		methyl 25-acetoxy-3β-hydroxycholest-5-en-19-carboxylate	-
**124**		11α-acetoxygorgostan-3β,5α,6β,12α-tetrol ((22*R*,23*R*)-23,24β-dimethyl-22,23-methano-11α-acetoxycholest-5-en-3β,5α,6β,12α-tetrol)	56962930
**125**		12α-acetoxygorgostan-3β,5α,6β,11α-tetrol ((22*R*,23*R*)-23,24β-dimethyl-22,23-methano-12α-acetoxycholestan-3β,5α,6β,11α-tetrol)	-
**126**		11α-acetoxygorgostan-3β,5α,6β,15α-tetrol ((22*R*,23*R*)-23,24β-dimethyl-22,23-methano-12α-acetoxycholestan-3β,5α,6β,15α-tetrol)	-
**127**		7β-acetoxyergosta-5,24(28)-dien-3β,19-diol 24-methylidene-7β-acetoxycholest-5-en-3β,19-diol	477494
**128**	halymeniaol	3β,15α,16β-triacetoxy-12β-hydroxycholest-5-en-7-one	-
**129**	21-*O*-octadecanoyl-xestokerol A	(20*S*,21*R*)-21-octadecanoyl-11β,20,22-trihydroxypetrostan-3-one ((20*S*,21*R*,25*R*,26*R*)-24α,26-dimethyl-26,27-cyclo-21-octadecanoyl-11β,20,22-trihydroxycholestan-3-one)	71747680
**130**	xestokerol A	(20*S*,21*R*)-11β,20,21,22-tetrahydroxypetrostan-3-one ((20*S*,21*R*,25*R*,26*R*)-24α,26-dimethyl-26,27-cyclo-11β,20,21,22-tetrahydroxycholestan-3-one)	44584465
**131**	xestokerol A dimethyl ketal	(20*S*,21*R*)-3,3-dimethoxypetrostan-11β,20,21,22-tetrol ((20*S*,21*R*,25*R*,26*R*)-24α,26-dimethyl-26,27-cyclo-3,3-dimethoxycholestan--11β,20,21,22-tetrol)	-
**132**	7α-hydroxypetrosterol	petrost-5-en-3β,7α-diol ((25*R*,26*R*)-24α,26-dimethyl-26,27-cyclocholest-5-en-3β,7α-diol)	101209535
**133**	7β-hydroxypetrosterol	petrost-5-en-3β,7β-diol ((25*R*,26*R*)-24α,26-dimethyl-26,27-cyclocholest-5-en-3β,7β-diol)	71747681
**134**	7-ketopetrosterol	3β-hydroxypetrost-5-en-7-one ((25*R*,26*R*)-24α,26-dimethyl-26,27-cyclo-3β-hydroxycholest-5-en-7-one)	101209534
**135**	petrosterol	petrost-5-en-3β-ol ((25*R*,26*R*)-24α,26-dimethyl-26,27-cyclocholest-5-en-3β-ol)	194249
**136**	11β-hydroxypetrosterol	petrost-5-en-3β,11βα-diol ((25*R*,26*R*)-24α,26-dimethyl-26,27-cyclocholest-5-en-3β,11β-diol)	-
**137**		(20*S*)-petrostan-3α,7α,12β,20-tetrol ((20*S*,25*R*,26*R*)-24α,26-dimethyl-26,27-cyclocholestan-3α,7α,12β,20-tetrol)	-
**138**		(20*S*)-petrostan-3α,12β,14α,20-tetrol ((20*S*,25*R*,26*R*)-24α,26-dimethyl-26,27-cyclocholestan-3α,12β,14α,20-tetrol)	-
**139**		(20*S*)-3,3-dimethoxypetrostan-7α,12β,20-triol ((20*S*,25*R*,26*R*)-24α,26-dimethyl-26,27-cyclo-3,3-dimethoxycholestan-7α,12β,20-triol)	-
**140**		(20*S*)-3,3-dimethoxypetrostan-7α,12β,19,20-tetrol ((20*S*,25*R*,26*R*)-24α,26-dimethyl-26,27-cyclo-3,3-dimethoxycholestan-7α,12β,19,20-tetrol)	-
**141**		(20*S*)-petrostan-3α,12β,20-triol ((20*S*,25*R*,26*R*)-24α,26-dimethyl-26,27-cyclocholestan-3α,12β,20-triol)	-
**142**		(20*S*)-petrostan-3β,12β,20-triol ((20*S*,25*R*,26*R*)-24α,26-dimethyl-26,27-cyclocholestan-3β,12β,20-triol)	-
**143**	aragusterol B	(20*S*)-12β,20-dihydroxypetrostan-3-one ((20*S*,25*R*,26*R*)-24α,26-dimethyl-26,27-cyclo-12β,20-dihydroxycholestan-3-one)	44566420
**144**		(20*S*)-7α,12β,20-trihydroxypetrostan-3-one ((20*S*,25*R*,26*R*)-24α,26-dimethyl-26,27-cyclo-7α,12β,20-trihydroxycholestan-3-one)	-
**145**		3,3-dimethoxypetrostan-12β,16α-diol ((25*R*,26*R*)-24α,26-dimethyl-26,27-cyclo-3,3-dimethoxycholestan-12β,16α-diol)	-
**146**	aragusterol A	(20*R*,22*S*)-20,21-epoxy-12β,22-dihydroxypetrostan-3-one ((20*R*,22S,25*R*,26*R*)-24α,26-dimethyl-20,21-epoxy-26,27-cyclo-12β,22-dihydroxycholestan-3-one)	9933873
**147**		(20*R*,22*S*)-20,21-epoxy-3,3-dimethoxypetrostan-12β,22-diol ((20*R*,22S,25*R*,26*R*)-24α,26-dimethyl-20,21-epoxy-26,27-cyclo-3,3-dimethoxycholestan-12β,22-diol)	10696885
**148**		(22*R*)-12β,22-dihydroxypetrost-20(21)-en-3-one ((22*R*,25*R*,26*R*)-24α,26-dimethyl-26,27-cyclo-12β,22-dihydroxycholest-20(21)-en--3-one)	-
**149**	aragusterol J	(22*R*)-7β,12β,22-trihydroxypetrost-20(21)-en-3-one ((22*R*,25*R*,26*R*)-24α,26-dimethyl-26,27-cyclo-7β,12β,22-trihydroxycholest-20(21)-en--3-one)	-
**150**	klyflaccisteroid C	3β,7α-dihydroxygorgost-5-en-11-one ((22*R*,23*R*)-23,24β-dimethyl-22,23-methano-3β,7α-dihyroxycholest-5-en-11-one)	-
**151**	klyflaccisteroid D	3β-hydroxygorgost-5-en-7,11-dione ((22*R*,23*R*)-23,24β-dimethyl-22,23-methano-3β,7α-dihyroxycholest-5-en-7,11-dione)	-
**152**	klyflaccisteroid E	gorgosta-5,9(11)-dien-3β,7β,12α-triol ((22*R*,23*R*)-23,24β-dimethyl-22,23-methanocholesta-5,9(11)-dien-3β,7β,12α-triol)	-
**153**		gorgost-5-en-3β,9α,11α-triol ((22*R*,23*R*)-23,24β-dimethyl-22,23-methanocholest-5,-dien-3β,9α,11α-triol)	10742556
**154**	klyfaccisteroid H	gorgost-5-en-3β,11α,12α-triol ((22*R*,23*R*)-23,24β-dimethyl-22,23-methanocholesta-5,9(11)-dien-3β,11α,12α-triol)	-
**155**	halistanol sulfate	24,25-dimethylcholestane-2β,3α,6α-trisulfate	73361
**156**	halistanol sulfate I	24-methyl-24,25-methanocholestane-2β,3α,6α-trisulfate	-
**157**	halistanol sulfate J	24,24-(methylethano)cholestane-2β,3α,6α-trisulfate	-
**158**	solomonsterol A	trisodium cholane-2β,3α,24-trisulfate (trisodium 25,26,27-trinorcholestane-2β,3α,24-trisulfate)	50925451
**159**	solomonsterol B	trisodium 24-norcholane-2β,3α,23-trisulfate (trisodium 24,25,26,27-tetranorcholestane-2β,3α,24-trisulfate)	53318073
**160**	theonellasterol	4-methylidineporiferast-8(14)-en-3β-ol (24β-ethyl-4-methylidinecholest-8(14)-en-3β-ol)	52931395
**161**	conicasterol	4-methylidinecampest-8(14)-en-3β-ol (24α-methyl-4-methylidinecholest-8(14)-en-3β-ol)	21670674
**162**	ganoderic acid A	7β,15α-dihydroxy-3,11,23-trioxolanost-8-en-26-oic acid 4α,4β,14α-trimethyl-7β,15α-dihydroxy-3,11,23-trioxocholest-8-en-26-oic acid)	471002
**163**		ergosta-7,9(11),22*E*-trien-3β-ol 24β-methylcholesta-7,9(11),22*E*-trien-3β-ol	12308954
**164**		ergosta-4,7,22*E*-trien-3-one 24β-methylcholesta-4,7,22*E*-trien-3-one	11003773
**165**		ergosta-4,6,8(14),22*E*-tetraen-3-one 24β-methylcholesta-4,6,8(14),22*E*-tetraen-3-one	6441416
**166**		14α-hydroxyergosta-4,7,9(11),22*E*-tetraen-3,6-dione 24β-methyl-14α-hydroxycholesta-4,7,9(11),22*E*-tetraen-3,6-dione	10251684
**167**		9α,14α-dihydroxyergosta-4,7,22*E*-trien-3,6-dione 24β-methyl-9α,14α-dihydroxycholesta-4,7,22*E*-trien-3,6-dione	-
**168**		ergosta-4,6,8(14),22*E*,24(28)-pentaen-3-one 24-methylidenecholesta-4,6,8(14),22*E*-tetraen-3-one	-
**169**	nodulisporiviridin E	18-nor-1α,3β-dihydroxy-4,5,6-[2,3,4]furanoandrosta-5,8,11,13(14)-tetraen-7,17-dione 18,20,21,22,23,24,25,26,27-nonanor-1α,3β-dihydroxy-4,5,6-[2,3,4]furanoandrosta-5,8,11,13(14)-tetraen-7,17-dione	122179368
**170**	nodulisporiviridin F	3β,11β-dihydroxy-4,5,6-[2,3,4]furanoandrosta-5,8-dien-7,17-dione 20,21,22,23,24,25,26,27-octanor-3β,11β-dihydroxy-4,5,6-[2,3,4]furanocholesta-5,8-dien-7,17-dione	122179369
**171**	nodulisporiviridin G	11β-hydroxy-4,5,6-[2,3,4]furanoandrosta-5,8-dien-3,7,17-trione 20,21,22,23,24,25,26,27-octanor-11β-hydroxy-4,5,6-[2,3,4]furanocholesta-5,8-dien-3,7,17-trione	122179370
**172**	nodulisporiviridin H	3β,12β-dihydroxy-4,5,6-[2,3,4]furanoandrosta-5,8-dien-7,17-dione 20,21,22,23,24,25,26,27-octanor-3β,12β-dihydroxy-4,5,6-[2,3,4]furanocholesta-5,8-dien-7,17-dione	122179371
**173**	16-*O*-desmethylasporyergosterol-β-d-mannoside	β-d-mannosyloxyergosta-6,8(14),17(20)*E*, 22*E*-tetraen-3β-ol 24β-methyl-β-d-mannosyloxycholesta-6,8(14),17(20)*E*,22*E*-tetraen-3β-ol	-
**174**		(24*S*)-24,28-epoxyergost-5-en-3β,4α-diol (24*S*)-24-oxyranylcholest-5-en-3β,4α-diol	44575614
**175**		ergosta-5,24(28)-dien-3β,7α-diol (24-methylidenecholest-5-en-3β,7α-diol)	10949727
**176**		ergosta-5,24(28)-dien-3β,7β-diol (24-methylidenecholest-5-en-3β,7β-diol)	11373355
**177**		ergost-5-en-3β,7β-diol (24β-methylcholest-5-en-3β,7β-diol)	11475561
**178**		ergost-24(28)-en-3β,5α,6β-triol (24-methylidenecholestan-3β,5α,6β-triol)	21775108
**179**		ergostan-3β,5α,6β-triol (24β-methylcholestan-3β,5α,6β-triol)	44558918
**180**		3β,5α,6β,11α-tetrahydroxyergostan-1-one (24β-methyl-3β,5α,6β,11α-tetrahydroxycholestan-1-one)	-
**181**		ergostan-1α,3β,5α,6β,11α-pentol (24β-methylcholestan-1α,3β,5α,6β,11α-pentol)	-
**182**	sarcoaldesterol B	ergostan-3β,5α,6β,11α-tetrol (24β-methylcholestan-3β,5α,6β,11α-tetrol)	10718409
**183**		ergostan-1β,3β,5α,6β-tetrol (24β-methylcholestan-1β,3β,5α,6β-tetrol)	-
**184**	pregnedioside A	4α-*O*-β-d-arabinopyranosyloxypregn-20-en-3β-ol22,23,24,25,26,27-hexanor-4α-*O*-β-d-arabinopyranosyloxycholest-20-en-3β-ol	21673267
**185**		gorgostan-1α,3β,5α,6β,11α-pentol ((22*R*,23*R*)-23,24β-dimethyl-22,23-methanocholestan-1α,3β,5α,6β,11α-pentol)	23426029
**186**	sarcoaldesterol A	gorgostan-3β,5α,6β,11α-tetrol ((22*R*,23*R*)-23,24β-dimethyl-22,23-methanocholestan-3β,5α,6β,11α-tetrol)	10790775
**187**		(20*R*,23*R*)-23-methylergost-16-en-3β,20-diol (20*R*,23*R*)-23,24β-methylcholest-16-en-3β,20-diol	-
**188**	ximaosteroid E	(16S)-16,22-epoxycholesta-1,22*E*-dien-3-one	-
**189**	ximaosteroid F	(20*R*,22*R*)-20,22-dihydroxycholesta-1,4-dien-3-one	-
**190**		(20*S*)-20-hydroxycholest-1-en-3,16-dione	53997071
**191**	sinubrasone A	methyl (22*R*)-22-*O*-β-d-xylopyranosyloxy-3-oxoergosta-1,4-diene-26-carboxylate methyl (22*R*)-24β-methyl-22-*O*-β-d-xylopyranosyloxy-3-oxocholesta-1,4-diene-26-carboxylate	-
**192**	sinubrasone B	methyl (16*S*,22*R*)-16-methoxy-16,22-epoxy-3-oxoergosta-1,4-diene-26-carboxylate methyl (16*S*,22*R*)-24β-methyl-16-methoxy-16,22-epoxy-3-oxocholesta-1,4-diene-26-carboxylate	-
**193**	sinubrasone C	methyl (22*R*,23*R*)-22,23-epoxy-3-oxoergosta-1,4-diene-26-carboxylate methyl (22*R*,23*R*)-24β-methyl-22,23-epoxy-3-oxocholesta-1,4-diene-26-carboxylate	-
**194**	sinubrasone D	methyl (20*S*)-20-methyl-3-oxopregna-1,4-diene-21-carboxylate methyl 23,24,25,26,27-pentanor-3-oxocholesta-1,4-diene-21-carboxylate	15929041
**195**		ergostan-1α,3β,5α,6β,11α,15α-hexol (24β-methylcholestan-1α,3β,5α,6β,11α,15α-hexol)	-
**196**		ergostan-3β,5α,6β,15α-tetrol (24β-methylcholestan-3β,5α,6β,15α-tetrol)	-
**197**		ergostan-3β,5α,6β,11α,15α-pentol (24β-methylcholestan-3β,5α,6β,11α,15α-pentol)	-
**198**		ergost-7-en-3β,5α,6β,15α-tetrol (24β-methylcholest-7-en-3β,5α,6β,15α-tetrol)	-
**199**		23-methylergost-22*E*-en-3β,5α,6β,11α-tetrol (23,24β-dimethylcholest-22*E*-en-3β,5α,6β,11α-tetrol)	-
**200**	klyflaccisteroid A	(17*S*,23*R*)-23-methylergosta-5,20(21)-dien-3β,17α-diol (17*S*,23*R*)-23,24β-dimethylcholesta-5,20(21)-dien-3β,17α-diol	-
**201**	klyfaccisteroid J	(20*R*,23*R*)-23-methylergosta-5,16-dien-3β,11α,20-triol (20*R*,23*R*)-23,24β-dimethylcholesta-5,16-dien-3β,11α,20-triol	-
**202**	klyflaccisteroid M	(22*S*)-ergost-5-en-3β,7β,22-triol ((22*S*)-24β-methylcholest-5-en-3β,7β,22-triol)	-
**203**	subergorgol U	19(10→4)-abeo-2-hydroxypregna-2,4,1(10)-trien-20-one (22,23,24,25,26,27-hexnor-19(10→4)-abeo-2-hydroxycholesta-2,4,1(10)-trien-20-one)	132918691
**204**		19(10→4)-abeo-1-hydroxypregna-2,4,1(10)-trien-20-one (22,23,24,25,26,27-hexnor-19(10→4)-abeo-2-hydroxycholesta-2,4,1(10)-trien-20-one)	54484024
**205**		(20*S*)-7α,12β,20-trihydroxycholest-22*E*-en-3-one	-
**206**	langcosterol A	26,27-dimethylergosta-5,24(28)-dien-3β,7α-diol (26,27-dimethyl-24-methylidenecholest-5-en-3β,7α-diol)	23426186
**207**		ergosta-4,7,22*E*,25-tetraen-3-one (24β-methylcholesta-4,7,22*E*,25-tetraen-3-one)	132280531
**208**		7α,12β,18-trihydoxystigmast-22*E*-en-3-one (24α-methyl-7α,12β,18-trihydoxycholest-22*E*-en-3-one)	-
**209**		(20*S*)-24-ethyl-7α,12β,20-trihydroxycholestan-3-one	-
**210**		(20*S*)-24-methyl-7α,12β,20-trihydroxycholest-22*E*-en-3-one	-
**211**	7,15-dioxoconicasterol	4-methylidene-3β-hydroxycampest-8(14)-en-7,15-dione (24α-methyl-4-methylidene-3β-hydroxycholest-8(14)-en-7,15-dione)	-
**212**	15-oxoconicasterol	4-methylidene-3β-hydroxycampest-8(14)-en-15-one (24α-methyl-4-methylidene-3β-hydroxycholest-8(14)-en-15-one)	-
**213**		4-methylidene-3β,9α-dihydroxycampest-8(14)-en-15-one (24α-methyl-4-methylidene-3β,9α-dihydroxycholest-8(14)-en-15-one)	-
**214**	gelliusterol E	24-methylchola-5,16-dien-23-yn-3β,7α-diol (26,27-dinorcholesta-5,16-dien-23-yn-3β,7α-diol)	-
**215**	saringosterol	24-ethenylcholest-5-en-3β,24-diol	14161394
**216**	dictyosterol A	3β,6β-dihydroxycholesta-4,22*E*-dien-24-one	-
**217**	dictyosterol B	6β-hydroxycholesta-4,22*E*-dien-3,24-dione	-
**218**	dictyosterol C	3β,7α-dihydroxycholesta-5,22*E*-dien-24-one	-
**219**		3β-hydroxycholesta-5,22*E*-dien-7,24-dione	-
**220**		3β-hydroxycholesta-5,22*E*-dien-24-one	-
**221**	dictyopterisin C	(24*R*)-stigmasta-4,28(29)-dien-3β,7β,24-triol (24*R*)-24-ethenylcholest-4-en-3β,7β,24-triol	-
**222**		(24*R*)-7-methoxystigmasta-4,28(29)-dien-3β,24-diol (24*R*)-7-methoxy-24-ethenylcholest-4-en-3β,24-diol	-
**223**	dictyopterisin F	(24*R*)-3β,24-dihydroxystigmasta-4,28(29)-dien-7-one (24*R*)-24-ethenyl-3β,24-dihydroxycholest-4-en-7-one	-
**224**	dictyopterisin G	(24*S*)-3β,24-dihydroxyporiferasta-4,28(29)-dien-7-one (24*S*)-24-ethenyl-3β,24-dihydroxycholest-4-en-7-one	-
**225**	dictyopterisin H	(24*R*)-stigmasta-4,28(29)-dien-3β,6β,24-triol (24*R*)-24-ethenylcholest-4-en-3β,6β,24-triol	-
**226**	dictyopterisin I	(24R)-6β,24-dihydroxystigmasta-4,28(29)-dien-3-one (24R)-24-ethenyl-6β,24-dihydroxycholest-4-en-3-one	-
**227**	dictyopterisin J	(24*S*)-6β,24-dihydroxyporiferasta-4,28(29)-dien-3-one (24*S*)-24-ethenyl-6β,24-dihydroxycholest-4-en-3-one	-

^1^ Compound number. ^2^ Systematic names use carbon numbering and side chain α/β designations of the Nes system presented in Figure 1 and Ref. [1].
